# Charcot–Marie–Tooth disease variants of HSPB1 progressively alter neuromuscular signalling

**DOI:** 10.1242/dmm.052805

**Published:** 2026-08-28

**Authors:** Iman Aolymat, Jeff W. Barclay

**Affiliations:** Department of Biochemistry, Cell and Systems Biology, ISMIB, University of Liverpool, Liverpool L69 3BX, UK

**Keywords:** Charcot–Marie–Tooth disease type 2, Hsp27, Synaptic transmission, Aldicarb

## Abstract

Autosomal dominant variants in HSPB1 can cause type 2 Charcot–Marie–Tooth disease, a progressive neuromuscular disorder. HSPB1 is a small, ATP-independent chaperone that functions in protein folding, stabilisation and stress protection, as well as regulating intracellular processes such as organization of the cytoskeleton. How diverse variants in HSPB1 exert progressive neuromuscular defects is unclear. Using a transgenic *Caenorhabditis elegans* model, we examined three distinct HSPB1 variants and determined that mutant HSPB1 caused an early defect in neuromuscular signalling strength as assayed by aldicarb sensitivity. Our data point to the effects being presynaptic in origin and unrelated to differences in expression of mutant HSPB1 compared with that of the wild-type protein. In contrast, the early defect in neuromuscular signalling may be a result of a general loss of small chaperone function. We also identified a progressive effect whereby age-dependent changes to neuromuscular signalling were accelerated in worms expressing mutant HSPB1. These effects were not mirrored by a general loss of small chaperone function; however, wild-type HSPB1 expression strikingly protected against progressive defects seen in controls. These results indicate a possible progressive mechanism for HSPB1-dependent neuromuscular defects.

## Teaser

**Summary:** Progressive changes in neuromuscular signalling are preserved by wild-type HSPB1, whereas Charcot–Marie–Tooth disease variants of HSPB1 decrease signalling in young animals and accelerate the aging process.

## INTRODUCTION

Charcot–Marie–Tooth disease (CMT) is a progressive, hereditary sensory and motor neuropathy of the peripheral nervous system (Laurá et al., 2019). The disease is the most commonly inherited neurological disorder and can cause variable symptoms from muscle weakness to foot deformities and distal muscle atrophy. CMT is highly heterogeneous; variants identified in over 100 genes have been associated with the disease with varying clinical severity (Pipis et al., 2019; Rossor et al., 2013). Broadly speaking, CMT is subdivided into two forms. CMT type 1 (CMT1) is well described as a demyelinating form caused by variants in Schwann cell proteins, resulting in a large decrease in peripheral axonal conduction velocity (Kamholz et al., 2000; Rossor et al., 2013). CMT type 2 (CMT2), in contrast, is defined as acting axonally, is less associated with a decrease in conduction velocity, and is connected with a large number of proteins involved in diverse axonal cellular processes such as mitochondrial dynamics, intracellular trafficking and mRNA processing (McCray and Scherer, 2021; Rossor et al., 2013). How variants in the underlying genes lead to clinical symptoms of CMT2 mechanistically is not always clearly defined. There is no known cure and available treatment is more palliative, aimed at managing symptoms. One of the possible reasons for this is the high heterogeneity of the disease and the potential broad underlying mechanisms that may be affected.

Heat shock protein beta-1 (HSPB1), also known as Hsp27, is one of the proteins in which variants can lead to CMT2 disease, as well as distal hereditary motor neuropathy (Vendredy et al., 2020) and amyotrophic lateral sclerosis (Capponi et al., 2016). HSPB1 is a member of the small heat shock protein (sHSP) family of ATP-independent holdase chaperones (Garrido et al., 2012; Gu et al., 2023). Expression of sHSPs in general, and HSPB1 specifically, is upregulated in response to cellular stress, such as heat shock, where it acts to prevent aggregation of unfolded proteins. The protein can exist in various oligomeric or dimeric forms; however, the active form of HSPB1 is thought to be the monomer (Almeida-Souza et al., 2010). In addition to neuroprotection, it is increasingly appreciated that constitutive levels of sHSPs have important endogenous functions in cells, including modulation of the cytoskeleton, cell signalling and apoptosis (Janowska et al., 2019). Over 40 distinct variants in HSPB1 have been associated with CMT2 (Abati et al., 2021); however, very few have any described associated mechanistic data.

Like most sHSPs, HSPB1 structurally consists of a conserved central α-crystallin domain flanked by variable N- and C-termini. In CMT2, variants in HSPB1 can occur anywhere in the protein structure (Abati et al., 2021), possibly predicting a heterogenic axonal mechanism. In concordance with this prediction, the S135F variant in the α-crystallin domain increases affinity for tubulin and chaperone activity, whereas the C-terminal P182L variant shows reduced affinity and no effect on chaperone ability (Almeida-Souza et al., 2011, 2010). It isn't clear, however, how these variable changes alter the physiology of the peripheral motor axon and, more importantly, how this exerts progressive defects. We investigated this question using transgenic expression of wild-type (WT) or CMT variants of HSPB1 in *Caenorhabditis elegans*. Irrespective of the structural location of the mutation, we found that a range of HSPB1 mutations caused an early-stage decrease in neuromuscular signalling strength. As the animal aged, however, transgenic expression of mutant HSPB1 prematurely aged the neuromuscular junction, as quantified by aldicarb sensitivity. The effects did not appear to be a consequence of postsynaptic changes nor were they wholly a consequence of a general loss of sHSP expression. These results present a possible mechanism underlying progressive defects associated with HSPB1-related CMT.

## RESULTS

### Background to CMT variants in HSPB1

**Fig. 1.**
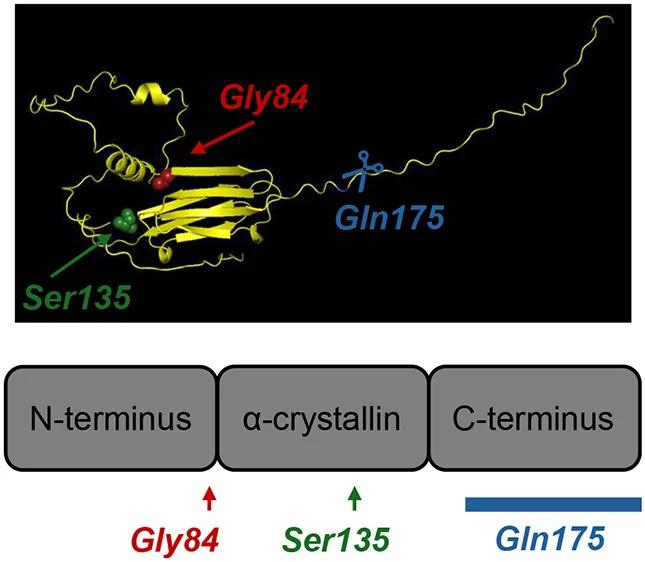
**Structural location of the wild-type residues for the Charcot–Marie–Tooth disease variants assessed in this study.** The top panel shows the predicted protein structure of human HSPB1 generated by AlphaFold (Fleming et al., 2025; Jumper et al., 2021) (accession number AF-P04792-F1-v6). The bottom panel shows a schematic outline of the coding structure for HSPB1. HSPB1 is broadly divided into three sections: a central, conserved α-crystallin domain flanked by two variable N- and C-termini. The Charcot–Marie–Tooth disease (CMT) variant of Gly84 (G84R) is located near the end of the N-terminal domain. The Ser135 variant (S135F) is located in the α-crystallin domain. Finally, the Gln175 variant is a nonsense mutation (Q175X) that truncates most of the C-terminus from the protein.

Variants in the human *HSPB1* gene underlie CMT type 2F, an autosomal dominant disease characterised by progressive distal muscle weakness, muscle atrophy, foot deformity and, to a lesser extent, sensory loss (Schwartz, 2019). We were interested in identifying progressive effects of HSPB1 variants at the neuromuscular junction. To this end, we specifically investigated three variants of HSPB1 associated with CMT, all of which are predicted to be pathogenic or likely pathogenic by ClinVar (https://www.ncbi.nlm.nih.gov/clinvar/). We selected example variants from each of the structural domains of the protein to study – one from the N-terminus of the protein (G84R), one in the conserved α-crystallin domain (S135F), and a truncation mutant of the C-terminus (Q175X) (Fig. 1). The S135F variant is one of the most common HSPB1 variants, presents with severe symptoms and is the best characterised experimentally. The variant is linked with increased monomerisation and an enhancement in thermotolerance (Almeida-Souza et al., 2010), a reduction in cell viability (Evgrafov et al., 2004), alterations to the cytoskeleton (Almeida-Souza et al., 2011; Zhai et al., 2007), trafficking defects (d'Ydewalle et al., 2011), mitochondrial impairment (Adriaenssens et al., 2023; Schwartz et al., 2018) and locomotor disability (Kang et al., 2020; Lee et al., 2015). Confusingly, despite being considered an axonal defect, HSPB1 S135F overexpressing transgenic mice also display aberrant myelination (Lee et al., 2015) indicating that there may be a myelination-dependent component to mutational effects. The N-terminal G84R variant is less characterised but has distinct effects including aggregation (James et al., 2008) and lower chaperone-like activity (Nefedova et al., 2013). Finally, the Q175X variant is a C-terminal truncation leaving the N-terminus and crystallin domain intact. Clinically, it presents as motor predominant (Echaniz-Laguna et al., 2017), but there have been no functional studies on this variant yet.

### CMT variants are transgenically expressed less than WT HSPB1

**Fig. 2.**
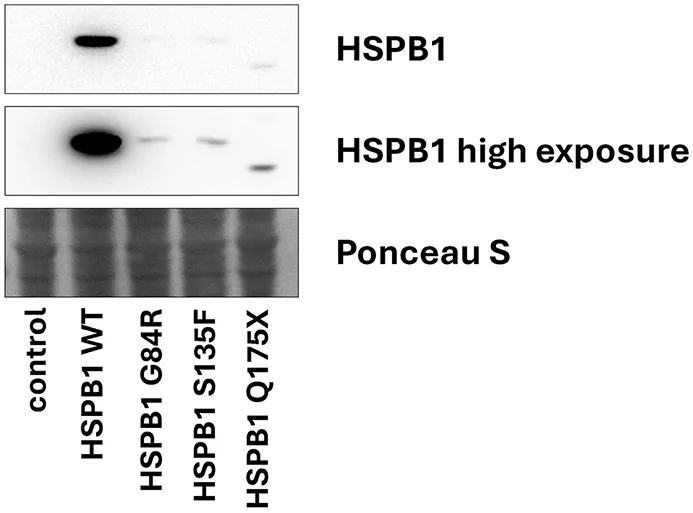
**Transgenic expression of HSPB1 constructs in *C. elegans*.** Western blot analysis showed that HSPB1 wild-type (WT) was more highly expressed in comparison to the three CMT variants (G84R, S135F and Q175X) in *C. elegans*. A higher-exposure blot demonstrated that all three variants are indeed expressed and that the truncation variant (Q175X) was of a smaller size as expected. Ponceau S staining shows equivalent loading of protein samples. The full, uncropped version of this blot can be found in Fig. S1.

HSPB1 variants are autosomal dominant; therefore, we utilised the *C. elegans* worm model to investigate the human *HSPB1* gene expressed transgenically in neurons. *C. elegans* do not have any axonal myelin, precluding any potential confounding role for myelination in the physiological effects. To this end, we created transgenic worms expressing WT or mutant HSPB1 under the control of a pan-neuronal promoter and assessed for neurological phenotypes that were conserved between the three distinct CMT-causing variants. To assess the extent of transgene expression, we performed western blotting on worm extracts from the transgenic strains (Fig. 2; Fig. S1). As expected, no signal for human HSPB1 was identified in the Bristol N2 controls, whereas a strong band was seen in the HSPB1 WT lane without any obvious cross-identification of endogenous α-crystallin domain-containing proteins. In contrast the three HSPB1 mutant variants were qualitatively equivalently expressed, but all at reduced levels in comparison to HSPB1 WT. As predicted, HSPB1 Q175X produced a truncated protein of ∼19 kDa, whereas the other two mutant proteins were full length.

### CMT variants in HSPB1 have variable effects on movement and chemosensation

**Fig. 3.**
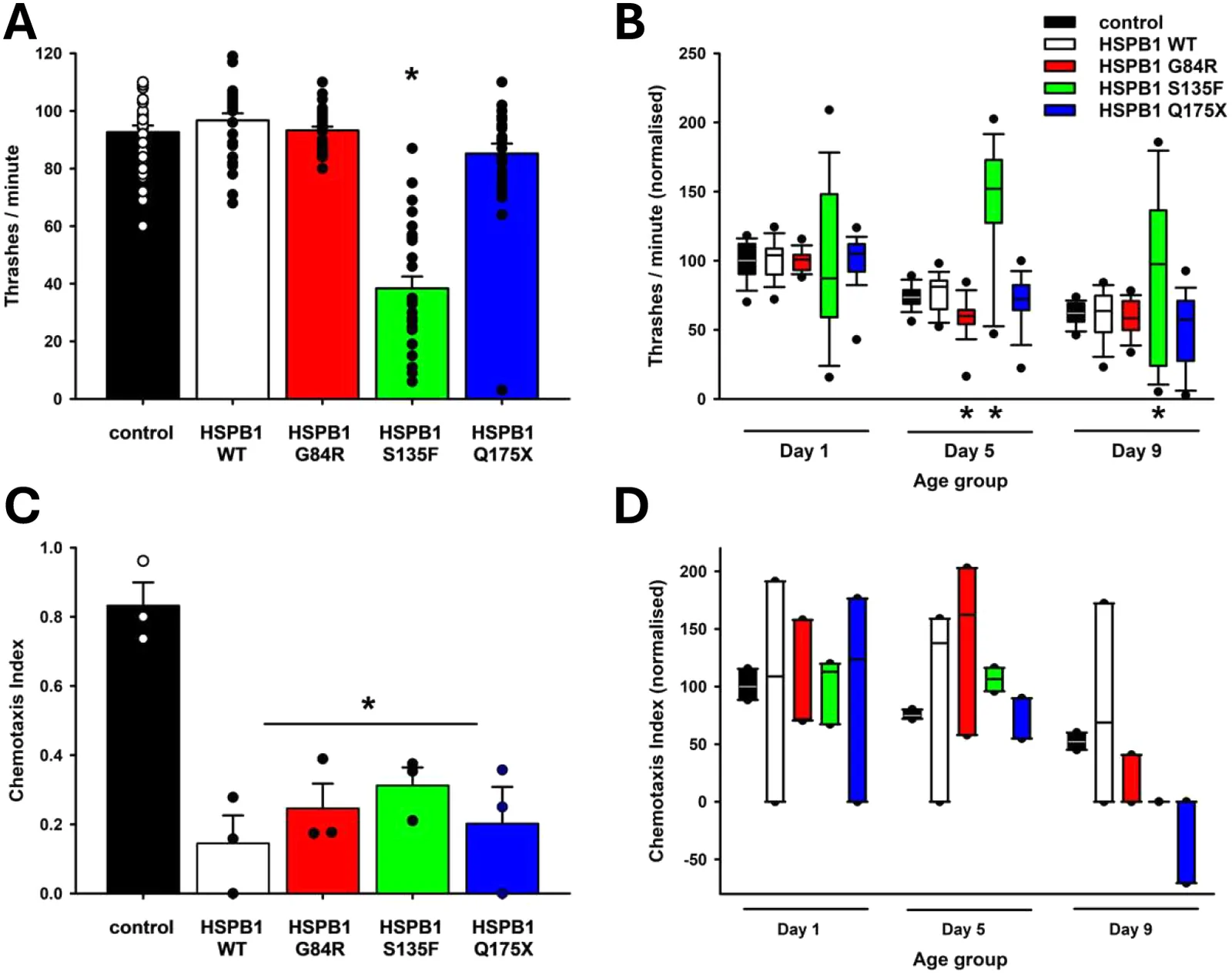
**Variable effects of HSPB1 CMT variants on *C. elegans* locomotion and chemotaxis.** (A) In comparison to Bristol N2 controls, transgenic expression of HSPB1 WT had no effect on worm locomotion, as assessed by counting thrashes per minute. Expression of HSPB1 S135F statistically reduced locomotion, whereas expression of the G84R and Q175X variants had no discernible effect. Datapoints represent individual animals. Data, shown as mean±s.e.m., were assessed by one-way ANOVA on ranks (H_4,30_=72.09, *P*<0.001) and Tukey's multiple comparison test compared to Bristol N2 controls (*P<0.05). (B) Progressively, locomotion of Bristol N2 controls decreased with age. Expression of HSPB1 WT showed no difference in progressive effects, whereas the expression of three examined HSPB1 variants had variable effects. Locomotion of G84R worms decreased at day 5, that of S135F worms increased at days 5 and 9, whereas locomotion of Q175X worms showed no difference from that of controls. Data were assessed by two-way ANOVA (F_4,30_=28.00, *P*<0.001) and Tukey's multiple comparison test compared to Bristol N2 controls (*P<0.05). Boxes show second and third quartiles; whiskers represent minimum and maximum values. (C) In comparison to Bristol N2 controls, expression of WT or all three variant HSPB1 constructs inhibited chemotaxis to 1-butanol. Data, shown as mean±s.e.m., were assessed by one-way ANOVA (F_4,3_=12.93, *P*<0.001) and Tukey's multiple comparison test compared to Bristol N2 controls (*P<0.05). (D) There were no statistically significant progressive effects for chemotaxis to 1-butanol for any worm strain examined. Data were assessed by two-way ANOVA (F_4,3_=0.92, *P*>0.05) compared to Bristol N2. Boxes show second and third quartiles; whiskers represent minimum and maximum values. The data presented in A and C were used to normalise progressive effects in B and D; however, original unnormalised data can be found in Fig. S2 (thrashing) and Fig. S3 (chemotaxis).

We next investigated whether there were any broad functional neuromuscular defects associated with HSPB1 expression by quantifying locomotion (Fig. 3A). For young adult *C. elegans* (day 1 of adulthood), expression of HSPB1 WT did not alter locomotion as assessed by thrashing in comparison with Bristol N2 controls. Individual expression of the HSPB1 variants exhibited conflicting outcomes on neuromuscular performance. Whereas the more clinically severe variant, S135F, caused a large reduction in locomotion, the other two variants had no obvious effect.

*C. elegans* have a short lifespan, allowing progressive changes to be quantified. As CMT is a progressive phenotype, we were specifically interested in identifying age-dependent changes in phenotype. To this end, we age-matched worms from a young age (adult age of 1 day) to an intermediate age (adult age of 5 days) and finally an older age (adult age of 9 days). Thrashing rates of worms decrease by age, dropping 30-40% in Bristol N2 controls by day 7-9 of adulthood (Kashyap et al., 2014; Mulcahy et al., 2013). In comparison to controls, expression of HSPB1 WT had no additional progressive effects on locomotion over the ages analysed (Fig. 3B; Fig. S2). Despite the association of all HSPB1 variants with progressive motor symptoms in patients, there was no obvious pattern evident when assessed in *C. elegans*. For the C-terminal truncation, Q175X, there was no statistical difference in locomotion to that for either HSPB1 WT or Bristol N2 controls. For the N-terminal missense variant, G84R, there was only a small depreciation in locomotion at day 5 that reverted to control levels at an older age. Finally, the effects of the crystallin domain variant, S135F, interestingly improved with age as a percentage of original locomotion rate. This enhancement was most likely a consequence of the S135F variant demonstrating a severe locomotion defect at a young age.

CMT is considered to have more motor than sensory involvement in clinical presentation. Nevertheless, we also investigated sensory defects in worms via a chemotaxis assay utilising 10^−3^ dilution of 1-butanol as a positive chemoattractant (Fig. 3C). During positive chemotaxis, animals utilise chemosensory neurons to recognise attractant molecules and then generate coordinated movement towards the attractant. In comparison to Bristol N2 controls, surprisingly, expression of any of the HSPB1 constructs (WT or mutant) negatively affected chemotaxis, as seen by worms randomly distributed on the chemotaxis analysis plates. It should be noted that, despite inherent differences in transgenic expression, the effects of the variants were not found to be any more severe than those of WT HSPB. Despite the negative impact of HSPB1 expression on chemotaxis, we also assessed for any progressive effects. Over the ages tested, there were no progressive effects on chemotaxis for Bristol N2 control worms (Fig. 3D; Fig. S3). There were also no progressive effects of HSPB1, WT or mutant, as chemotaxis remained ablated at all ages tested.

### CMT variants in HSPB1 reduce aldicarb sensitivity in young worms and accelerate progressive effects

**Fig. 4.**
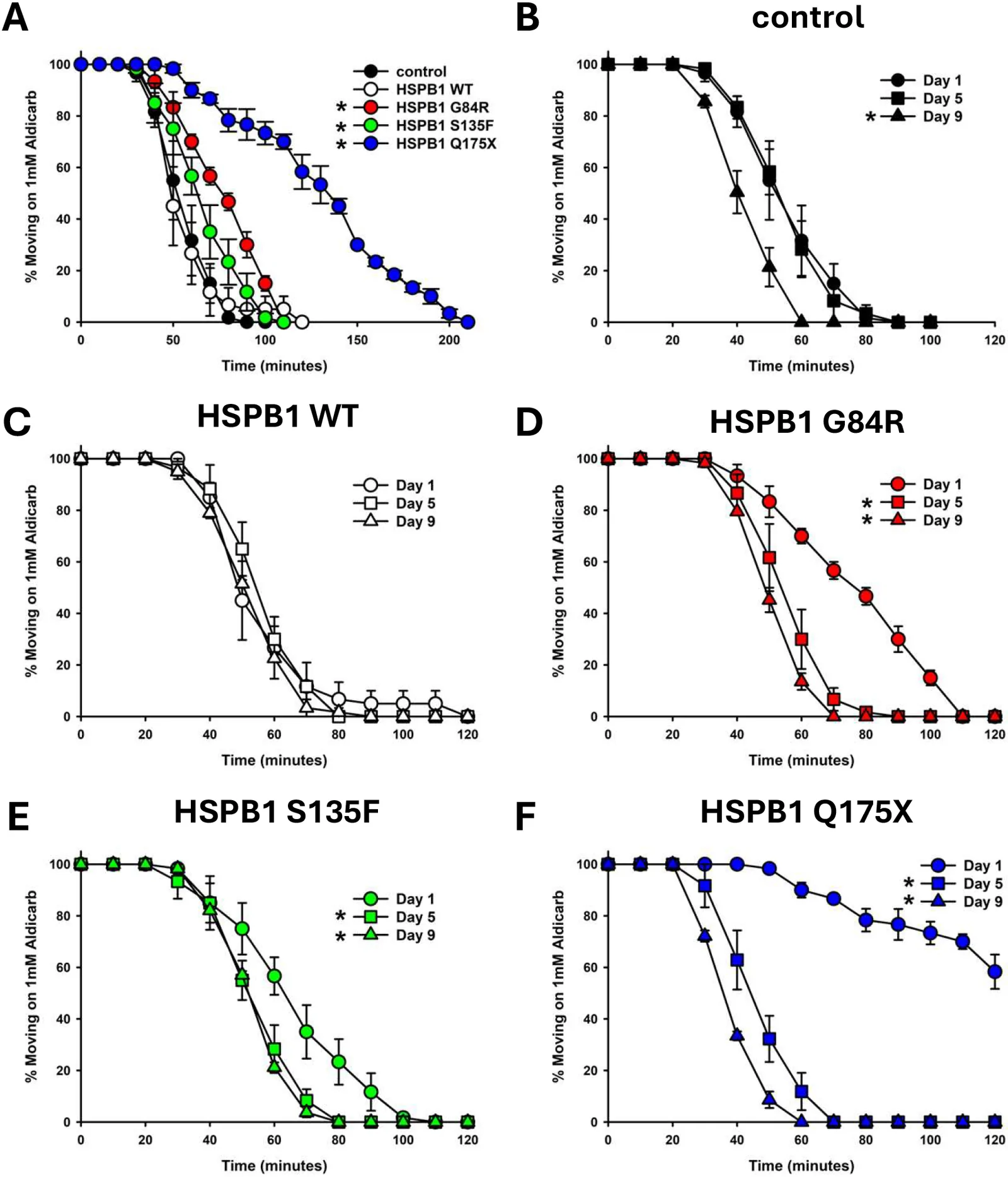
**Progressive effects of HSPB1 expression on aldicarb sensitivity in *C. elegans*.** (A) At a young age, expression of HSPB1 CMT variants caused resistance to aldicarb-induced paralysis in comparison to the behaviour of Bristol N2 controls. HSPB1 WT had no effect on aldicarb sensitivity. Data, shown as mean±s.e.m., were assessed by two-way ANOVA (F_4,3_=244.14, *P*<0.001) and Tukey's multiple comparison test compared to Bristol N2 controls (*P<0.05). (B) Bristol N2 controls showed a progressive shift towards aldicarb hypersensitivity between days 5 and 9 of adulthood (*P<0.05). (C) In comparison, HSPB1 WT-expressing worms were resistant to the progressive effects on aldicarb sensitivity over the ages examined. (D-F) Expression of individual CMT variants of HSPB1 induced aldicarb hypersensitivity at an earlier age than that in Bristol N2 controls. Each of G84R, S135F and Q175X HSPB1-expressing worms became hypersensitive to aldicarb between days 1 and 5 of adulthood (*P<0.05 for each). For B-F, data are shown as mean±s.e.m. and were assessed by two-way ANOVA (control: F_2,3_=15.896, *P*<0.001; WT: F_2,3_=1.66, *P*=0.20; G84R: F_2,3_=105.61, *P*<0.001; S135F: F_2,3_=18.48, *P*<0.001; Q175X: F_2,3_=1335.10, *P*<0.001) and Tukey's multiple comparison test compared to aldicarb sensitivity at day 1 (*P<0.05). The data presented in A were used for progressive comparisons in B-F.

To quantify neuromuscular signalling in nematodes directly, we utilised the aldicarb assay in which mutations that reduce neurotransmitter release make worms resistant to aldicarb and mutations that increase neurotransmitter release make worms hypersensitive (Mahoney et al., 2006). Expression of HSPB1 WT in nematodes did not affect aldicarb sensitivity, whereas worms expressing HSPB1 with CMT variants were all resistant to aldicarb (Fig. 4A). Interestingly, the crystallin-domain containing S135F variant, which is described as a more severe HSPB1 variant, exhibited the weakest phenotype, whereas the truncated Q175X variant had the strongest phenotype.

We also measured progressive effects on neuromuscular signalling. As has been previously demonstrated, Bristol N2 control worms become hypersensitive to aldicarb as they age (Johnson and Barclay, 2023; Mulcahy et al., 2013). In our experiments, we found that the shift to hypersensitivity for Bristol N2 controls occurred between days 5 and 9 of adulthood (Fig. 4B). Expression of HSPB1 WT, however, was associated with a complete resistance to the change towards hypersensitivity over the ages examined (Fig. 4C). In contrast, for worms expressing any of the three CMT HSPB1 variants, the progressive move towards hypersensitivity was evident at an earlier age than controls (Fig. 4D-F). Although all worms expressing the variants exhibited resistance to aldicarb at a young age, by day 5, this had already reversed to hypersensitivity.

### Decrease in sHSP expression phenocopies some aspects of CMT variants in HSPB1

**Fig. 5.**
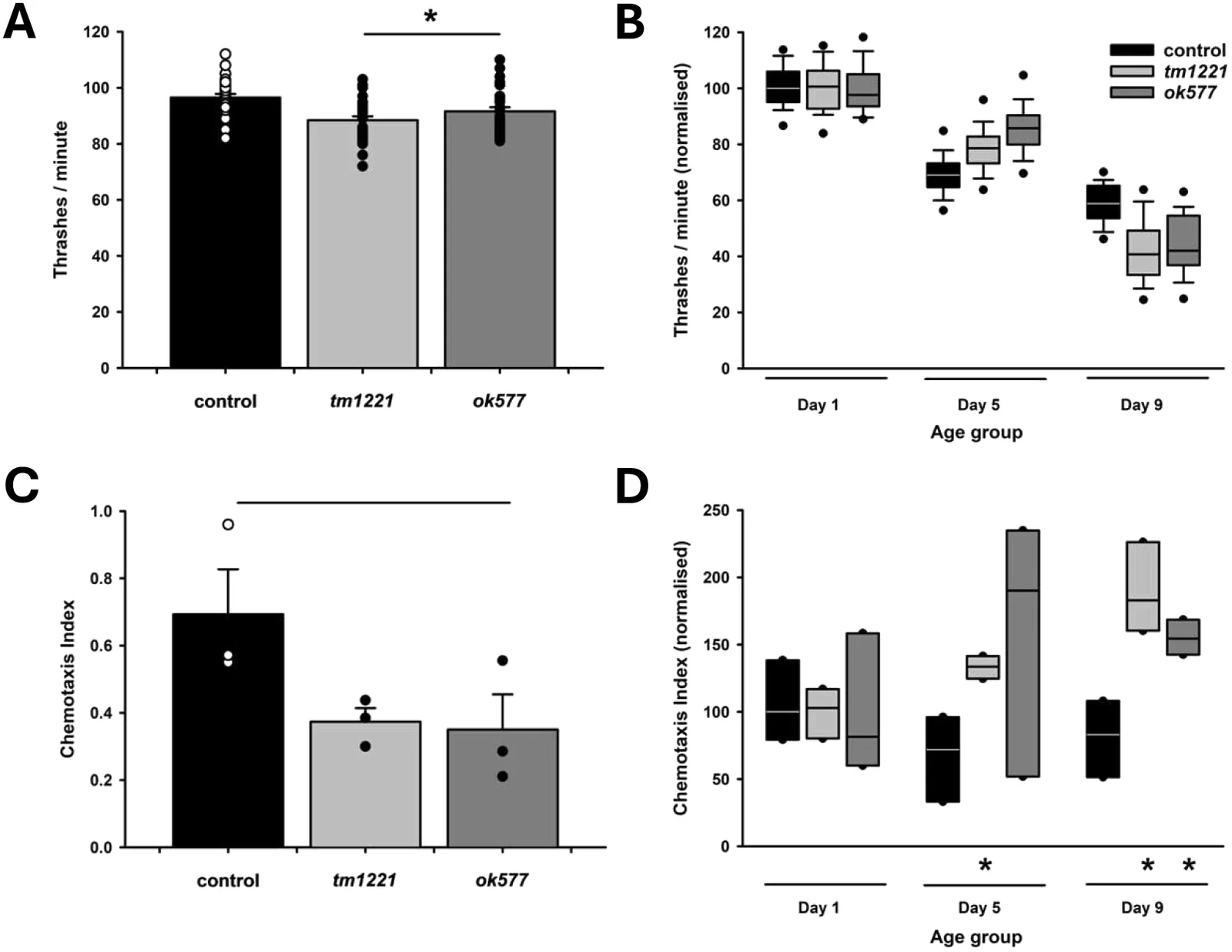
**Nominal effects of sHSP mutant strains on *C. elegans* locomotion and chemotaxis.** (A) In comparison to Bristol N2 controls, either the *ok577* allele (deletion mutation for genes *hsp-16.1/hsp-16.48*) or the *tm1221* allele (deletion mutation for the identical genes *hsp-16.11/hsp16.49*) showed minor reductions in thrashing rates. Data, shown as mean±s.e.m., were assessed by one-way ANOVA (F_2,30_=8.70, *P*<0.001) and Tukey's multiple comparison test compared to Bristol N2 controls (*P<0.05). (B) In comparison to Bristol N2 controls, there were no progressive effects on locomotion. Data were assessed by two-way ANOVA (F_2,30_=2.99, *P*>0.05) compared to Bristol N2 controls. Boxes show second and third quartiles; whiskers represent minimum and maximum values. (C) Both the sHSP mutant strains had a qualitative, but statistically insignificant, impact on chemotaxis to 1-butanol at a young age. Data, shown as mean±s.e.m., were assessed by one-way ANOVA (F_2,3_=3.64, *P*>0.05). Lack of significance is indicated by the straight black line above the graph. (D) Both the sHSP mutant strains showed an improvement in progressive chemotaxis. Data were assessed by two-way ANOVA (F_2,3_=4.87, *P*=0.02) and Tukey's multiple comparison test compared with Bristol N2 controls (*P<0.05). Boxes show second and third quartiles; whiskers represent minimum and maximum values. The data presented in A and C were used to normalise progressive effects in B and D; however, original unnormalised data can be found in Fig. S5 (thrashing) and Fig. S6 (chemotaxis).

HSPB1 is well characterised as a small ATP-independent chaperone and variants in CMT type 2F act dominantly. One possible explanation for the phenotypic effects seen here is a general decrease in small-chaperone activity in neurons, and this hypothesis could be tested experimentally by examining loss-of-function alleles of specific, endogenous sHSPs. It is inherently very difficult, however, to predict which nematode sHSP(s) might interact with HSPB1. sHSPs are classically defined by their conserved, central α-crystallin domain with variable N- and C-termini. As such, HSPB1 could theoretically interact with any of the 16 known α-crystallin domain-containing proteins in *C. elegans* (Fig. S4). To this end, we decided to test two worm alleles (*ok577* and *tm1221*): *ok577* is predicted to knock down expression of *hsp-16.48* and *hsp-16.1*, and *tm1221* is predicted to knock down expression of *hsp-16.49* and *hsp-16.11* (Johnson et al., 2016). These two alleles were selected as they both affect more than one sHSP with distinct, characterised worm phenotypes. *hsp-16.48* and *hsp-16.49* have been linked to neuroprotection from alcohol exposure (Johnson et al., 2016, 2017), whereas *hsp-16.1* and *hsp-16.11* are related to thermoprotection (Kourtis et al., 2012). We compared the *ok577* allele that knocks out half of these sHSPs (*hsp16.48* and *hsp-16.1*) and the *tm1221* allele that knocks out the other half (*hsp-16.49* and *hsp-16.11*). In terms of locomotion, there were only minimal effects of either of the sHSP knockdown strains at an early age and no additional progressive effects (Fig. 5A,B; Fig. S5). There were, however, variable effects on chemotaxis. At a young age, for both the *ok577* and the *tm1221* alleles, chemotaxis was qualitatively reduced but statistically similar to that in Bristol N2 controls (Fig. 5C). As the animals aged, however, worms with both the *tm1221* and *ok577* alleles became better at chemotaxis, whereas Bristol N2 controls remained relatively unaffected (Fig. 5D; Fig. S6).

**Fig. 6.**
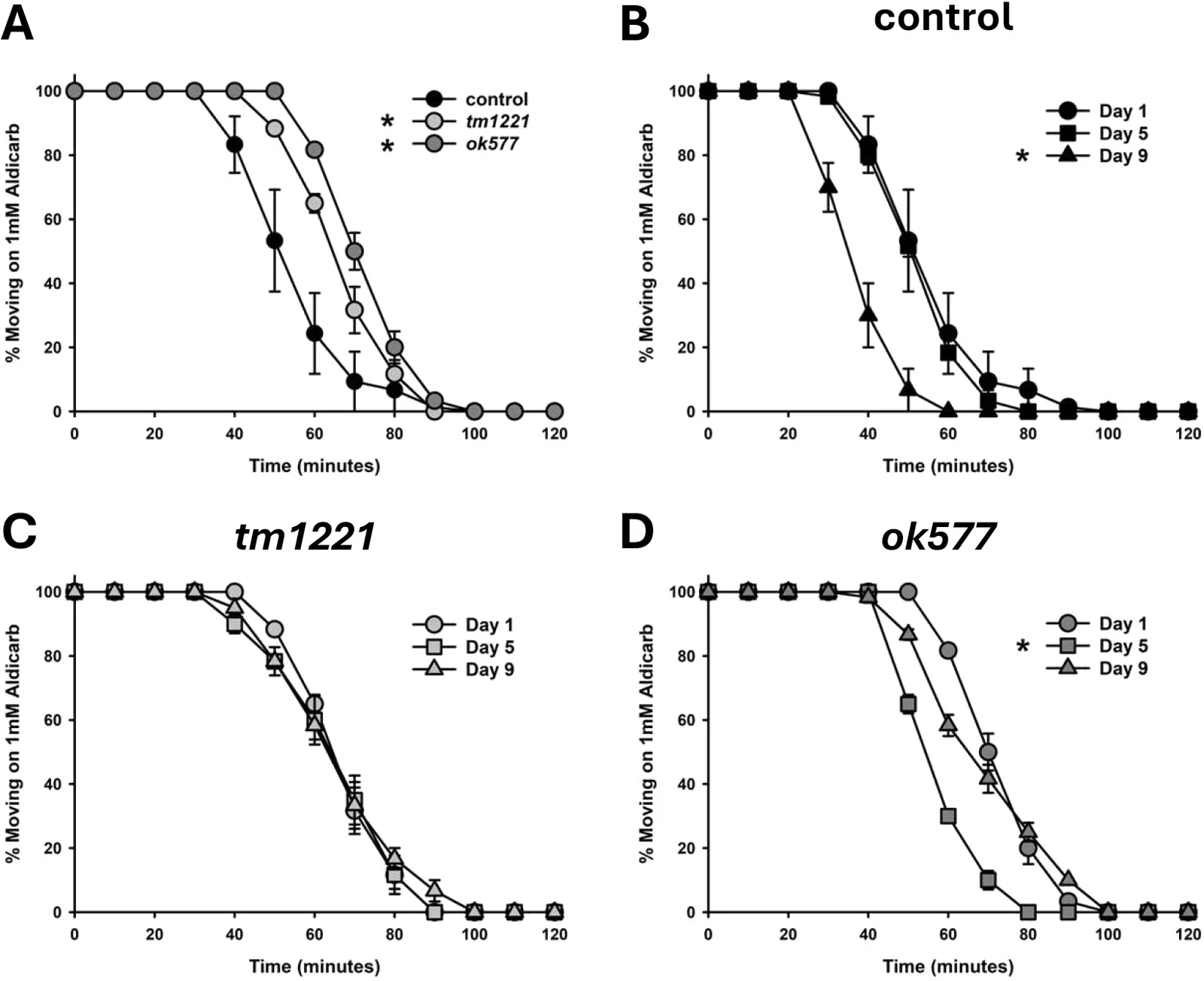
**Progressive effects of sHSP mutations on aldicarb sensitivity in *C. elegans*.** (A) Reminiscent of the mutant HSPB1 expression aldicarb phenotype, both the *ok577* allele (deletion mutation for genes *hsp-16.1/hsp-16.48*) and the *tm1221* allele (deletion mutation for the identical genes *hsp-16.11/hsp16.49*) were resistant to aldicarb-induced paralysis at a young age. Data, shown as mean±s.e.m., were assessed by two-way ANOVA (F_2,3_=31.57, *P*<0.001) and Tukey's multiple comparison test compared to Bristol N2 controls (*P<0.05). (B) Bristol N2 controls again showed a progressive shift towards aldicarb sensitivity between days 5 and 9 of adulthood (*P<0.05). (C,D) In contrast to the mutant HSPB1 expression progressive aldicarb phenotypes, both the sHSP mutant strains were resistant to the progressive effects on aldicarb sensitivity over the ages examined. The *ok577* allele had no effect on aldicarb sensitivity, whereas the *tm1221* allele became hypersensitive by day 5 (*P<0.05) and then reverted to being resistant by day 9. For B-D, data are shown as mean±s.e.m. and were assessed by two-way ANOVA (control: F_2,3_=29.72, *P*<0.001; *tm1221*: F_2,3_=0.93, *P*=0.4; *ok577*: F_2,3_=147.80, *P*<0.001) and Tukey's multiple comparison test compared to aldicarb sensitivity at day 1 (*P<0.05). The data presented in A were used for progressive comparisons in B-D.

Both the sHSP knockdown strains were resistant to aldicarb at a young age (Fig. 6A). This was reminiscent but less severe than that seen with some of the HSPB1 CMT variants. In direct contrast to these variants, however, aldicarb sensitivity for both the *ok577* and *tm1221* alleles remained relatively unaffected by age in comparison with that for the Bristol N2 controls (Fig. 6B-D). The *ok577* allele first became resistant to aldicarb and then reverted to being hypersensitive as it aged, whereas the *tm1221* allele was completely unaffected by age. The effects of age on aldicarb sensitivity for the Bristol N2 controls were similar to those seen previously. We conclude therefore that, although the initial resistance to aldicarb of the HSPB1 variants at a young age may be a consequence of a general decrease in small-chaperone activity, the progressive results do not fit with this hypothesis.

### CMT variants in HSPB1 do not broadly change levamisole sensitivity

**Fig. 7.**
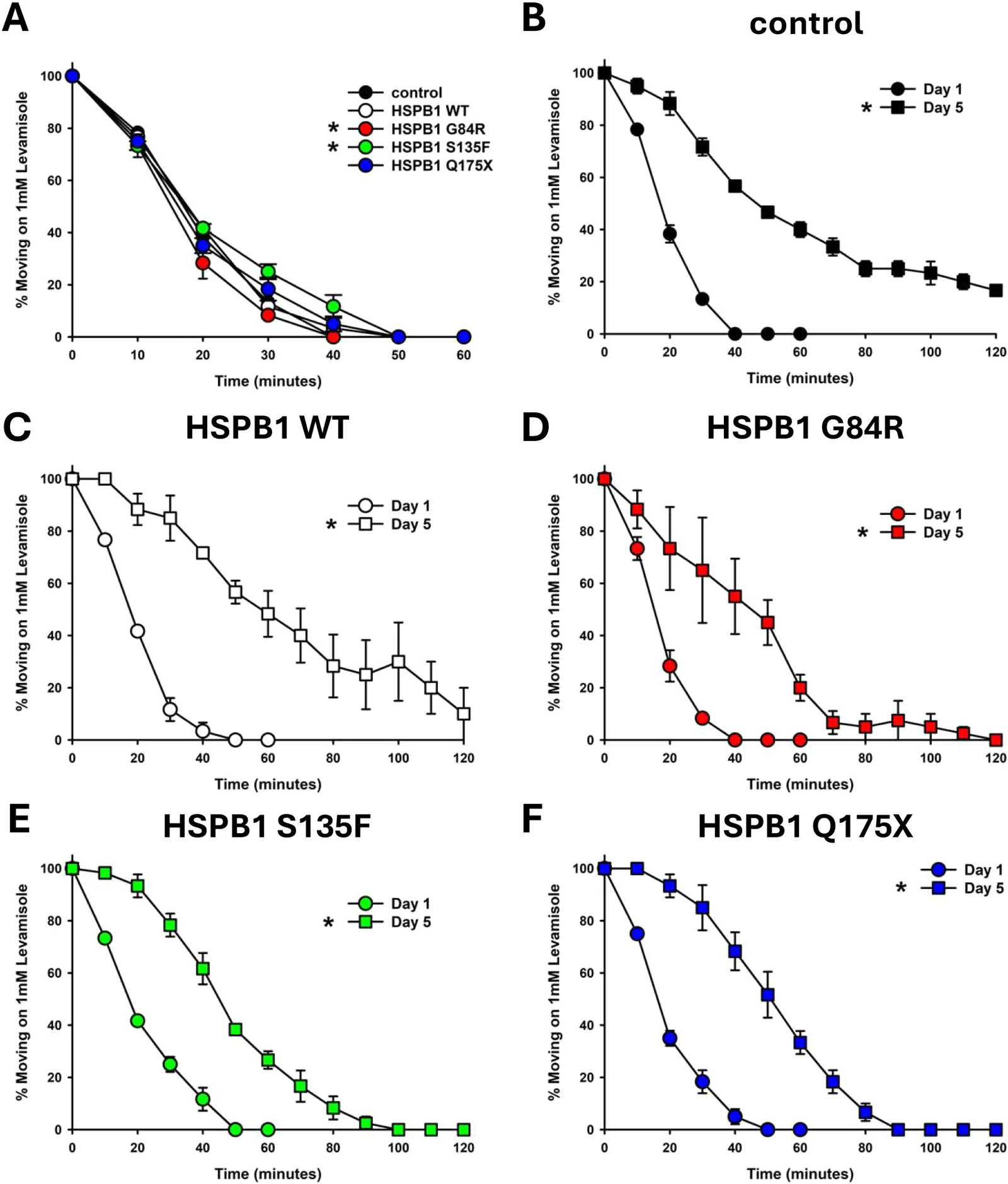
**Progressive effects of HSPB1 expression on levamisole sensitivity in *C. elegans*.** (A) At a young age, expression of HSPB1 WT or the Q175X variant had no effect on levamisole sensitivity, whereas the G84R variant was mildly hypersensitive and the S135F variant was mildly resistant. Data, shown as mean±s.e.m., were assessed by two-way ANOVA (F_4,3_=11.29, *P*<0.001) and Tukey's multiple comparison test compared to Bristol N2 controls (*P<0.05). (B) Bristol N2 controls showed a progressive shift towards levamisole resistance between days 1 and 5 of adulthood (*P<0.05). Expression of HSPB1 WT (C) or mutants (D-F) all showed the same progressive shift towards levamisole with age (*P<0.05 for each). For B-F, data are shown as mean±s.e.m. and were assessed by two-way ANOVA (control: F_1,3_=1372.68, *P*<0.001; WT: F_1,3_=205.88, *P*<0.001; G84R: F_1,3_=57.34, *P*<0.001; S135F: F_1,3_=402.56, *P*<0.001; Q175X: F_1,3_=336.04, *P*<0.001) and Tukey's multiple comparison test compared to levamisole sensitivity at day 1 (*P<0.05). The data presented in A were used for progressive comparisons in B-F.

Finally, we tested whether the progressive effects on aldicarb sensitivity of worms expressing mutant HSPB1 were the result of a postsynaptic change in neurotransmitter receptor sensitivity. This was quantified by the levamisole assay, in which levamisole acts as a direct nicotinic acetylcholine receptor agonist, bypassing possible effects on presynaptic release (Mahoney et al., 2006). In contrast to the effects of aldicarb treatment, at a young age, there were no consistent effects of HSPB1 expression on levamisole sensitivity for either WT or mutant HSPB1 (Fig. 7A). Although the HSPB1 G84R-expressing strain was mildly hypersensitive to levamisole and the HSPB1 S135F-expressing strain was mildly resistant, the HSPB1 WT- and HSPB1 Q175X-expressing strains were unaffected. Progressively, Bristol N2 control worms became resistant to the effects of levamisole by day 5 of adulthood (Fig. 7B). Similar to that seen for the young age, and in contrast to the aldicarb experiments, the progressive effects of HSPB1 WT or mutants on levamisole sensitivity were completely intact (Fig. 7C-F). These results are consistent with the effects of HSPB1 being presynaptic in origin.

## DISCUSSION

In this work, we identified the progressive effects of CMT variants of HSPB1 on neuromuscular signalling. To address axonal-specific effects distinct from myelination, we utilised a *C. elegans* transgenic model in which motor neurons are not insulated with myelin. Although expression of human HSPB1 WT had no effect on neuromuscular signalling at a young age, it protected against the age-dependent hypersensitivity to aldicarb evident in Bristol N2 control worms. In contrast, expression of three distinct CMT variants of HSPB1 showed an initial reduction in neuromuscular signalling at a young age and a progressive change to aldicarb hypersensitivity at an earlier age than controls. The effects seen at a younger age were consistent with a general reduction in sHSP function in worms; however, the later-age progressive effects appeared to be independent. Levamisole experiments supported the interpretation of presynaptic defects associated with transgenic HSBP1 expression. In total, these data support a progressive alteration in neuromuscular signalling strength associated with variant HSPB1 and potentially point towards an underlying factor in CMT pathophysiology.

### Differences in transgenic protein expression

All three distinct HSPB1 variants were expressed at similar levels, but less than was seen for HSPB1 WT. The exact underlying mechanism for this discrepancy remains unclear. HSPB1 itself is known to regulate gene expression, mRNA stability and translation (Cuesta et al., 2000; Fu et al., 2024); therefore, direct effects on transcription and/or translation machinery may be involved. Many point mutations and deletions can have large effects on protein folding and/or stabilisation, leading to reduced expression and compromised function (Backwell and Marsh, 2022). Misfolded proteins can be actively refolded or removed via various protein degradation pathways such as the ubiquitin-proteasome system. As proteins from the heat shock protein family are involved in both refolding and protein degradation (Fernández-Fernández et al., 2017), it is perhaps not surprising that variants in HSPB1 could affect these processes. Although HSPB1 G84R or Q175X expression levels have not previously been quantified, a reduction in HSPB1 S135F expression levels is not evident at least when expressed in transfected N2A cells (Evgrafov et al., 2004). It remains possible that the reduced expression observed here is a differentiated neuronal- or worm-specific outcome. Additionally, although the anti-HSPB1 antibody clearly recognises the variant forms and produces no off-target binding at the concentrations used, it cannot be conclusively ruled out that antibody affinities are not affected by the three selected HSPB1 variants. It also remains unclear whether there is an expression threshold that controls the phenotype and/or whether that threshold varies for HSPB1 WT and variants. Finally, the presented western blots were produced using mixed-age populations of worms. Given the progressive nature of the disease, it would be interesting to quantify the expression of protein as the animal ages. A thorough investigation of all of the potential mechanisms underlying differences in expression would be required to discriminate between all of these possible outcomes.

In any case, the phenotypic effects evident in variant HSPB1-expressing worms compared to HSPB1 WT are unlikely to be solely the result of excessive transgenic expression in a nematode model, as the WT protein had no effect on thrashing and aldicarb or levamisole sensitivities at young ages, despite a much greater level of expression. Despite creating the transgenic animals using the same constructs and promoters at the same expression level, given the experimental design of extrachromosomal arrays, it cannot be conclusively ruled out that the three individual HSPB1 variants do not lead to increased mosaicism or expression in non-neuronal cells. It is interesting to note, however, that the deleterious effects of transgenic HSPB1 expression on chemotaxis appear independent of extent of expression, where worms with higher-expressing HSPB1 WT were as negatively affected as those with lower-expressing HSPB1 variants.

### Effects of HSPB1 on *C. elegans* phenotypes

Given the extent of progressive phenotypic experiments analysed here, we only quantified single transgenic lines for each HSPB1 variant and, therefore, variations in transgenic expression between these individual lines cannot be ruled out. In addition to quantifying the protein expression levels, the experimental plan was to identify phenotypes that were similar for each of the three discrete HSPB1 variants. Our rationale was that a phenotype conserved between all three HSPB1 variants that cause a similar disease phenotype were more likely to be biologically relevant to the disease itself. We show here that there were indeed consistent variant phenotypes with respect to transgenic protein expression, namely reduced sensitivity to aldicarb at day 1, enhanced sensitivity to aldicarb progressively and, to a lesser extent, a lack of effect on levamisole sensitivity.

As our experiments transgenically expressed the human HSPB1 protein in *C. elegans*, the functional outcome of its variants is unlikely to be to be a simple loss-of-function phenotype as HSPB1 is not normally expressed in nematodes. HSPB1, and sHSPs in general, are well described as protein chaperones, in association with other sHSPs, ATP-dependent large HSPs or client proteins (Garrido et al., 2012; Gu et al., 2023; Janowska et al., 2019). It is thus likely that the variant HSPB1 protein would act in a dominant-negative manner via altered interactions with endogenous worm HSPs and/or client proteins; however, more general dominant neomorphic effects of the transgenic proteins cannot be excluded.

In contrast to the mutations, expression of HSPB1 WT had no effect on thrashing or aldicarb and levamisole sensitivities at an early age. This lack of phenotype could reflect the lack of any dominant-negative effects via endogenous worm sHSPs or client proteins or, again, a lack of general neomorphic effects for the WT protein. Expression of HSPB1 WT did, however, preserve aldicarb sensitivity over the ages investigated here. Given the well-established function for chaperones in ageing and proteostasis, this outcome is perhaps unsurprising. Future experiments will be required to tease apart the underlying mechanisms of these transgenic proteins in *C. elegans*.

### Comparing effects of CMT variants with loss of sHSP expression at a young age

Consistent with a dominant-negative hypothesis for endogenous worm HSPs, the initial effects of variant HSPB1 expression on aldicarb sensitivity were phenocopied by the loss-of-function alleles of endogenous sHSPs, at least at a young age. From the data, it is unclear which or both of the sHSPs *hsp-16.1* and *hsp-16.48* are responsible for the phenotype. Both *hsp-16.1* and *hsp-16.48* have been evolutionarily duplicated and reinserted in the genome, producing the identical genes *hsp-16.11* and *hsp-16.49*, respectively. Both of these genes progressively affect *C. elegans* lifespan and, like most characterised heat shock proteins, their expression is upregulated in response to heat stress (Hsu et al., 2003). Interestingly, only *hsp-16.1* and *hsp-16.11* are characterised as a chaperone associated with temperature-dependent stress (Kourtis et al., 2012), whereas *hsp-16.48* and *hsp-16.49* are associated with alcohol-dependent stress specifically (Johnson et al., 2016), emphasising some degree of functional divergence between the proteins. The *ok577* and *tm1221* alleles reciprocally delete one copy of each sHSP duplication – *hsp-16.1* and *hsp-16.48* (*ok577*), and *hsp-16.11* and *hsp-16.49* (*tm1221*) (Johnson et al., 2016). Therefore, it is unsurprising that the two mutations had mostly analogous phenotypes, where both mutant alleles demonstrated an initial reduction in thrashing, chemotaxis and a resistance to aldicarb.

### Comparing progressive effects of CMT variants with loss of sHSP expression

Although mostly consistent with a dominant-negative hypothesis at a young age, the phenotypes of the loss-of-function alleles of endogenous sHSPs did not support this hypothesis for progressive effects of transgenic HSPB1 expression. Both loss-of-function alleles of endogenous sHSPs had phenotypes more reminiscent of the HSPB1 WT expression rather than variant HSPB1 expression. It remains possible that the effects of expression of variant HSPB1 are distinct from the effects of loss of expression of sHSPs. This may be because variant HSPB1 primarily interacts with one or many of the other α-crystallin domain-containing proteins in the nematode or that the extent of loss of function for these alleles is not sufficient to phenocopy. Alternatively, it certainly remains possible that the investigated endogenous sHSPs are incompletely orthologous for HSPB1 or, indeed, that the correct ortholog for HSPB1 is any of the other sHSPs. A complete genetic analysis would be required to determine which sHSP is the correct functional ortholog for HSPB1, assuming it exists. Equally, the lack of a progressive negative impact in the sHSP aldicarb experiments fits with a progressive impact of the variant HSPB1 on client proteins other than via endogenous sHSPs. Finally, it cannot be ruled out that the variant HSPB1 expression could have a more widespread effect on endogenous nematode chaperones than is seen for the *ok577* or *tm1221* alleles or a more general dominant neomorphic effect.

### Potential site of action of HSPB1 variants

Results from the aldicarb and levamisole experiments suggest that the effects of HSPB1 variants are presynaptic in origin, rather than postsynaptic. This is perhaps not surprising given that the HSPB1 constructs were expressed specifically in neurons. It should be emphasised, however, that both aldicarb and levamisole effects do not conclusively identify location of action. Indeed, postsynaptic variants can alter aldicarb sensitivity and presynaptic variants can alter levamisole sensitivity (Mahoney et al., 2006). Alternatively, although the HSPB1 constructs were expressed pan-neuronally, any effects specific to GABAergic inhibitory motor neurons could also alter aldicarb sensitivity (Vashlishan et al., 2008). Finally, expression of certain chaperone proteins in worms can be regulated cell-non-autonomously via transcellular chaperone signalling (van Oosten-Hawle et al., 2013), and a similar effect cannot be ruled out here. Further genetic analysis into the precise location of action of the HSPB1 variants would be required to determine this point definitively.

### Potential underlying mechanisms of HSPB1 variants

How would HSPB1 functionally affect neuromuscular signalling? A number of different possibilities exist. HSPB1 is a well-described stabiliser of the axonal cytoskeleton, interacting in particular with tubulin as well as actin filaments and neurofilaments (Almeida-Souza et al., 2011; Clarke and Mearow, 2013; Zhai et al., 2007). Although *C. elegans* do not express neurofilaments *per se*, they do express intermediate filaments in neurons that fulfil a similar functional role (Zuela and Gruenbaum, 2016). Specific point variants of HSPB1 can alter binding to cytoskeletal components. For example, the S315F variant of HSPB1 enhances binding to tubulin, leading to microtubule network stabilisation (Almeida-Souza et al., 2011) and axonal transport deficits (d'Ydewalle et al., 2011). Although this enhancement in binding is not apparent for all CMT variants (e.g. the C-terminal P182L variant), it is possible that the three variants investigated here act by the overstabilisation of the axonal cytoskeleton, thus negatively impacting either axonal structure or trafficking. Alternatively, sHSPs may regulate neuromuscular signalling indirectly. HSP-16.1 is localised to the Golgi where it interacts with the Ca^2+^ and Mg^2+^-transporting ATPase PMR-1 to regulate calcium homeostasis in response to heat stress (Kourtis et al., 2012). Alterations to calcium levels at the neuromuscular junction could theoretically alter neurotransmitter release. Both HSP-16.1 and HSP-16.48 also directly bind the nematode Argonaute homolog ALG-1 (Chan and Slack, 2009), which could implicate a role for sHSPs in translational control of other integral neuronal proteins. Finally, neuromuscular signalling may be affected via a direct interaction with exocytosis proteins. Loss-of-function variants in most exocytotic proteins (SNAREs, Rabs, etc.) cause resistance to the aldicarb phenotype (Barclay et al., 2012; Mahoney et al., 2006), reminiscent of the variant HSPB1 effects seen here. Interestingly, a different chemosensory phenotype in worms showed that mutation or knockdown of the endogenous sHSP encoded by *hsp-16.48* is genetically phenocopied by a phosphorylation mutation of the essential exocytotic protein encoded by *unc-18* (Johnson et al., 2017). Although direct interactions with HSPBs have not yet been identified, the chaperone role of HSPB1 could be involved in facilitating protein–protein interactions that underpin synaptic exocytosis.

### Negative impact of transgenic HSPB1 expression on chemotaxis

In addition to neuromuscular signalling, transgenic HSPB1 expression, either WT or mutant, negatively affected chemotaxis. Amphid neurons, and the AWC^OFF^ neuron specifically for 1-butanol, are necessary for chemosensation in *C. elegans* (Bargmann et al., 1993; Brown et al., 2025). The neuronal pathway for chemotaxis requires G protein-coupled receptor signalling via cGMP-gated channels, eventually impacting upon motor neurons and controlling worm locomotion (Bargmann, 2006). From the data, it is unknown at what level HSPB1 expression negatively effects chemotaxis to 1-butanol. It is unlikely that the ablation of chemotaxis was the consequence of differential expression or effects on axonal trafficking or synaptic transmission, as both WT and mutant HSPB1 completely ablated chemotaxis at all ages. Given the limited impact of HSPB1 expression on worm locomotion, it is more likely that the negative impact of chemotaxis occurred at the level of the amphid neurons themselves or the processing interneurons prior to motor neurons. In these sites, HSPB1 itself could negatively impact expression of proteins integral to chemosensation or regulate the cGMP intracellular signalling pathway. In support of this hypothesis, mutation of *hsp-16.48* in IL2 chemosensory neurons specifically controls an ethanol-dependent increase in locomotion (Johnson et al., 2017) although data from both the *ok577* and *tm1221* alleles do not correspond phenotypically. Both of these alleles showed a slight, albeit non-significant, inhibition of chemotaxis to 1-butanol at a young age and an improvement in chemotaxis with age. The exact underlying role for transgenic HSPB1 in *C. elegans* chemotaxis remains to be determined.

HSPBs are relatively small, compact proteins consisting of a conserved, central α-crystallin domain flanked by variable N- and C-termini. Variants in HSPB1 linked to CMT2 can be found in any of these three domains and it is unknown what discrepancies in protein conformation might arise from any of these variants. Our data show that, despite the differences in structural location, all three variants caused a number of similar phenotypic outcomes (a reduction in expression, an initial resistance to aldicarb and an increase in the progressive sensitivity to aldicarb) and indicate that a general alteration in HSPB1 function may underlie the disease. This transgenic *C. elegans* model is a valuable tool to investigate progressive changes in whole-animal physiology *in vivo*, independent of potential alterations to myelination. Given the limited treatment options for patients with CMT, these worm strains could be very useful as platforms to identify and characterise genetic or pharmacological modifiers to the progressive effects of HSPB1 variants.

## MATERIALS AND METHODS

### Molecular biology

A mammalian expression plasmid for human HSPB1 was acquired from Addgene (plasmid #63092; deposited by Harm Kampinga). The coding region for *HSPB1* was first subcloned into pDONR201 (Invitrogen) using the following primers: HSPB1 attB fwd, 5ʹ-GGGGACAAGTTTACAAAAGCAGGCTTCATAGACCGAGCGCCGCGGTC-3ʹ; and HSPB1 attb rev, 5ʹ-GGGGACCACTTTGTACAAGAAAGCTGGGTCAGATTACTTGGCGGCAGTCTCATTCGG-3ʹ.

Mutagenesis of HSPB1 ENTRY clones was performed using the QuikChange II Site-Directed Mutagenesis Kit (Agilent Technologies) following the manufacturer's protocol and validated by Sanger sequencing. HSPB1 constructs were then recombined into a P*rab-3* DEST vector (a kind gift from Michael Nonet, Washington University, St. Louis, MO, USA) for neuronal-specific nematode expression, as previously described (Johnson et al., 2016).

### Nematode strains and culture

Worms were cultured on nematode growth medium (NGM) agar plates at 20°C with *Escherichia coli* OP50 as a food source (Brenner, 1974; Graham et al., 2009). The following strains were used in this study: Bristol N2 (control), RB791 [*hsp-16.1* (*ok577*) *hsp-16.48* (*ok577*)] and FX1221 [*hsp-16.11* (*tm1221*) *hsp-16.49* (*tm1221*)]; mutant alleles were back-crossed into N2 worms four times. An evolutionary duplication event has copied the *hsp-16.1/hsp-16.48* sequence, inverted and re-inserted it downstream in chromosome V as *hsp-16.11/hsp-16.49*. Both the *ok577* and *tm1221* alleles are small deletions that completely remove one copy of each sHSP, leaving the other untouched (Johnson et al., 2016). As such, they would be predicted to act as expression knockdown controls for each other and we have characterised them specifically to identify phenotypes that are consistent to both strains.

Transgenic worms expressing either WT or mutant HSPB1 were made by standard DNA microinjection into the germline and by creation of stable extrachromosomal arrays (Rieckher and Tavernarakis, 2017). Extrachromosomal arrays of exogenous DNA are inherited in a non-Mendelian fashion and were identified by a co-transformation marker. 20 ng/μl of the generated expression Gateway DEST vectors with a pan-neuronal promoter (*Prab-3::HSPB1 WT*, *Prab-3::HSPB1 G84R*, *Prab-3::HSPB1 S135F* or *Prab-3::HSPB1 Q175X*) was injected alongside 25 ng/μl of a *Psur-5::mCherry* co-expression marker into Bristol N2 controls (SUNY Biotech, Fuzhou, China). For each transgenic strain, at least three individual independently derived lines were isolated. These lines were first analysed in preliminary experiments for phenotypic consistency, before taking one line forward for full age-dependent characterisation. Following this, any phenotypic effects consistent for each of the discrete HSPB1 variants were considered more likely to be a potential mutant HSPB1 effect. The transgenic strains quantified were AMG635 (*Bristol N2; Ex[Prab-3::HSPB1 WT Psur-5::mCherry]*), AMG641 (*Bristol N2; Ex[Prab-3::HSPB1 G84R Psur-5::mCherry]*), AMG647 (*Bristol N2; Ex[Prab-3::HSPB1 S135F Psur-5::mCherry]*) and AMG653 (*Bristol N2; Ex[Prab-3::HSPB1 Q175X Psur-5::mCherry]*).

### Western blotting

Worm strains were grown on NGM plates seeded with OP50 as a food source and collected as mixed-age samples in nuclease-free water. Worm lysates were mixed with an equal volume of 2× Laemmli sample buffer and frozen at −80°C. Following three freeze-thaw cycles, samples were then boiled and separated on NuPAGE Bis-Tris gels (Thermo Fisher Scientific) and transferred to nitrocellulose. Transferred proteins were stained for total protein with Ponceau S solution before immunoblotting with 0.5 μg/ml anti-Hsp27 (Abcam, ab2790).

### Phenotypic analysis

For phenotypic experiments, *C. elegans* strains were age-synchronised by bleaching as previously described (Barker et al., 2022). Briefly, populated plates of adult worms were washed and incubated in two parts 8% commercial hypochlorite bleach and one part 5 M NaOH. Eggs were released by intermittent vortexing for 10 min, washed in distilled H_2_O and then pipetted onto freshly seeded NGM plates to grow until the L4 stage was reached. Age synchronisation was maintained by passaging worms onto fresh NGM plates daily until required for analysis. Experiments were performed on young (defined here as adult day 1), intermediate (adult day 5) or old (adult day 9) hermaphrodites. All assays were assessed visually without masking sample identities, utilising different populations of animals per age group. Locomotion was assessed by thrashing assay in 200 μl Dent's solution (140 mM NaCl, 6 mM KCl, 1 mM CaCl_2_, 1 mM MgCl_2_ and 5 mM HEPES, pH 7.4) supplemented with 0.1 mg/ml bovine serum albumin (Graham et al., 2009; Johnson et al., 2016). Worms were assessed in individual 35-mm Petri dishes and thrashes per minute were calculated over a 1 min recording time following 10 min acclimation in Dent's solution. One thrash was defined as one complete movement from maximum to minimum amplitude and back. Thrashing assays were conducted on ten individual worms per replicate with three biologically independent replicates in total per age. The chemotaxis assay was conducted based on previously described methods (Bargmann et al., 1993; Kashyap et al., 2014). Prior to analysis, worms were first moved onto a clear, unseeded NGM plate for 1 h, before placing them on chemotaxis analysis plates [9-cm Petri dishes containing 2% (w/v) agar, 5 mM KH_2_PO_4_, 1 mM CaCl_2_ and 1 mM MgSO_4_] prepared in advance. On opposite ends of the plate, 2 μl of 10^−3^ dilution of 1-butanol (diluted in ethanol) was applied as a positive chemoattractant with distilled H_2_O as control. 4 μl of 50 μg/ml sodium azide was also added to each end of the plate to paralyse worms after the chemoattractant or control was reached. Chemotaxis index was measured by subtracting the number of worms at the control spot away from the number of worms at the test spot and then dividing by the total number of moved worms. The assay was conducted on a minimum of 30 worms per replicate with three replicates in total per age. Acute sensitivity to aldicarb (Sigma-Aldrich) or levamisole (Sigma-Aldrich) was assessed by time to paralysis of a population of worms on an unseeded NGM agar plate supplemented with 1 mM aldicarb or levamisole, as previously described (Edwards et al., 2012; Mahoney et al., 2006). For both, paralysis was assessed every 10 min following exposure to the drug and was defined as a failure to respond to a light mechanical stimulation. The assays were conducted on a minimum of 20 individual worms per replicate with three replicates per age.

### Statistical analysis

Data were visualised and analysed in SigmaPlot 15 (Grafiti). Significance was assessed by one- or two-way ANOVA with Tukey’s post hoc test for multiple comparisons.

## Supplementary Material

10.1242/dmm.052805_sup1 Supplementary information
