# Supplementary material for: Charcot–Marie–Tooth disease variants of HSPB1 progressively alter neuromuscular signalling
Source: Dis Model Mech. 2026 Aug 28;19(8):dmm052805. doi: 10.1242/dmm.052805 (PMC13580553; doi:10.1242/dmm.052805)
Supplement: Supplementary file 1 [file dmm-19-052805-s1.pdf]

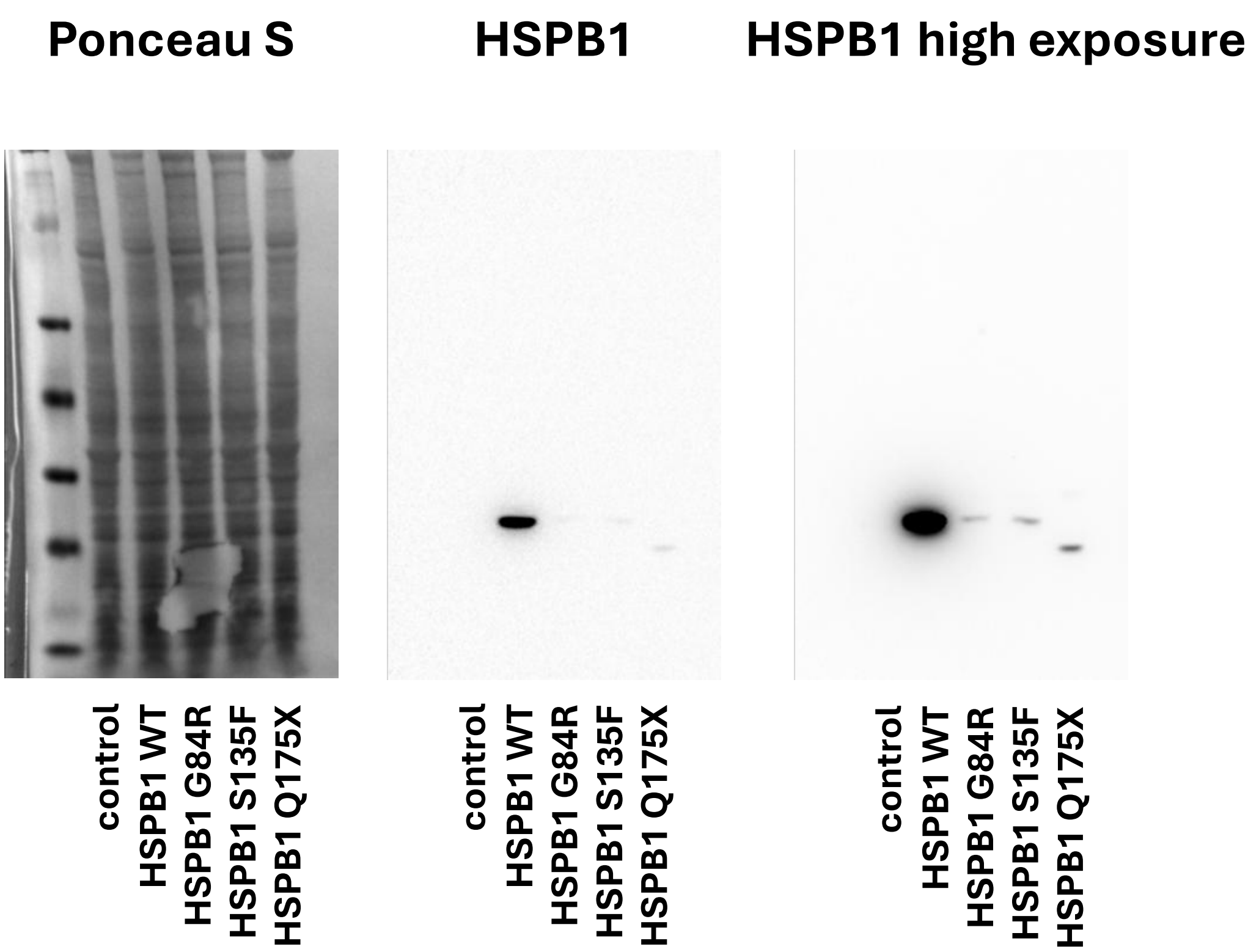

Fig. S1. Transgenic expression of HSPB1 constructs in *C. elegans*. Full western blots and Ponceau stains for qualitative protein expression comparisons.

Disease Models & Mechanisms • Supplementary information

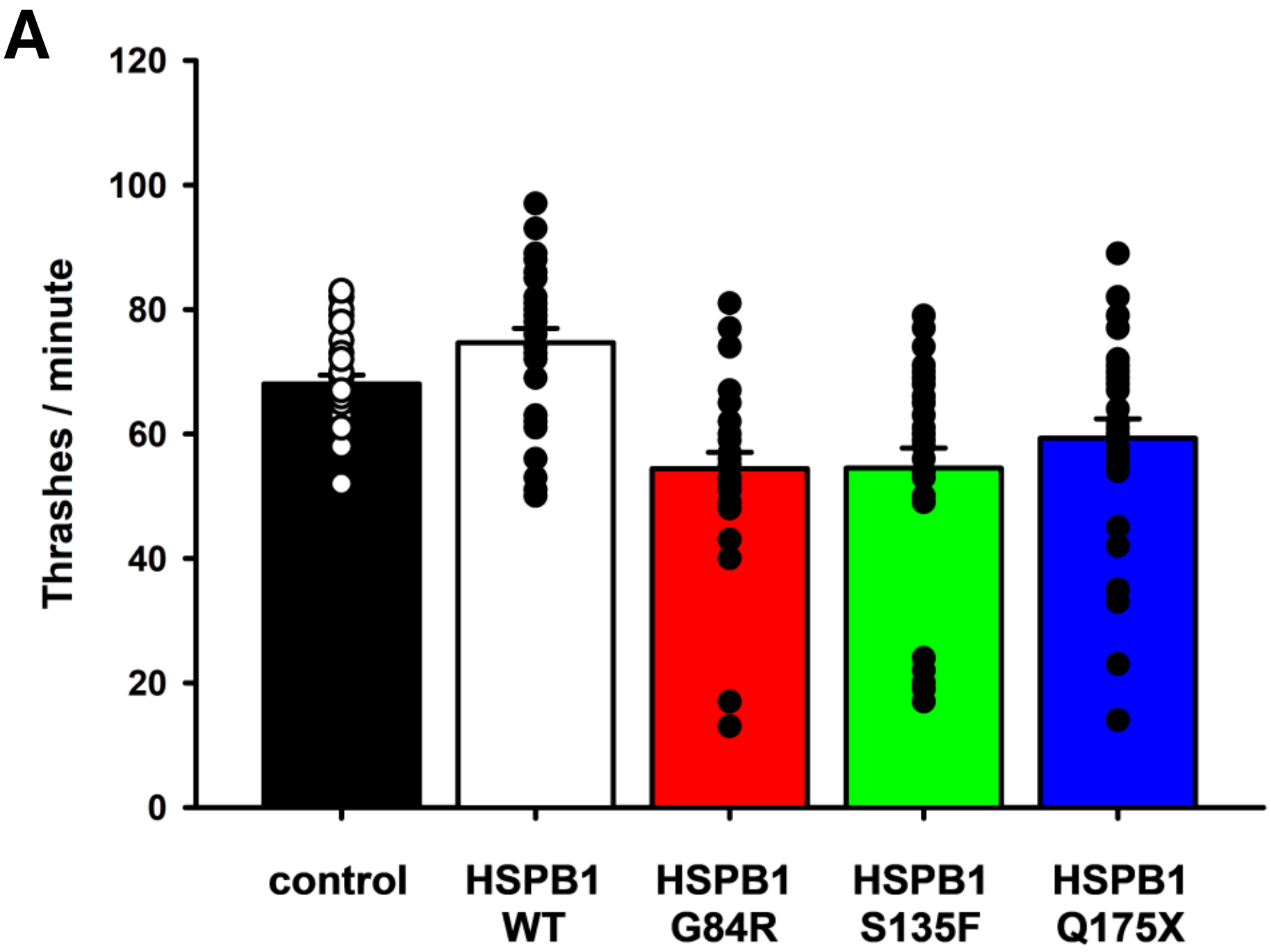

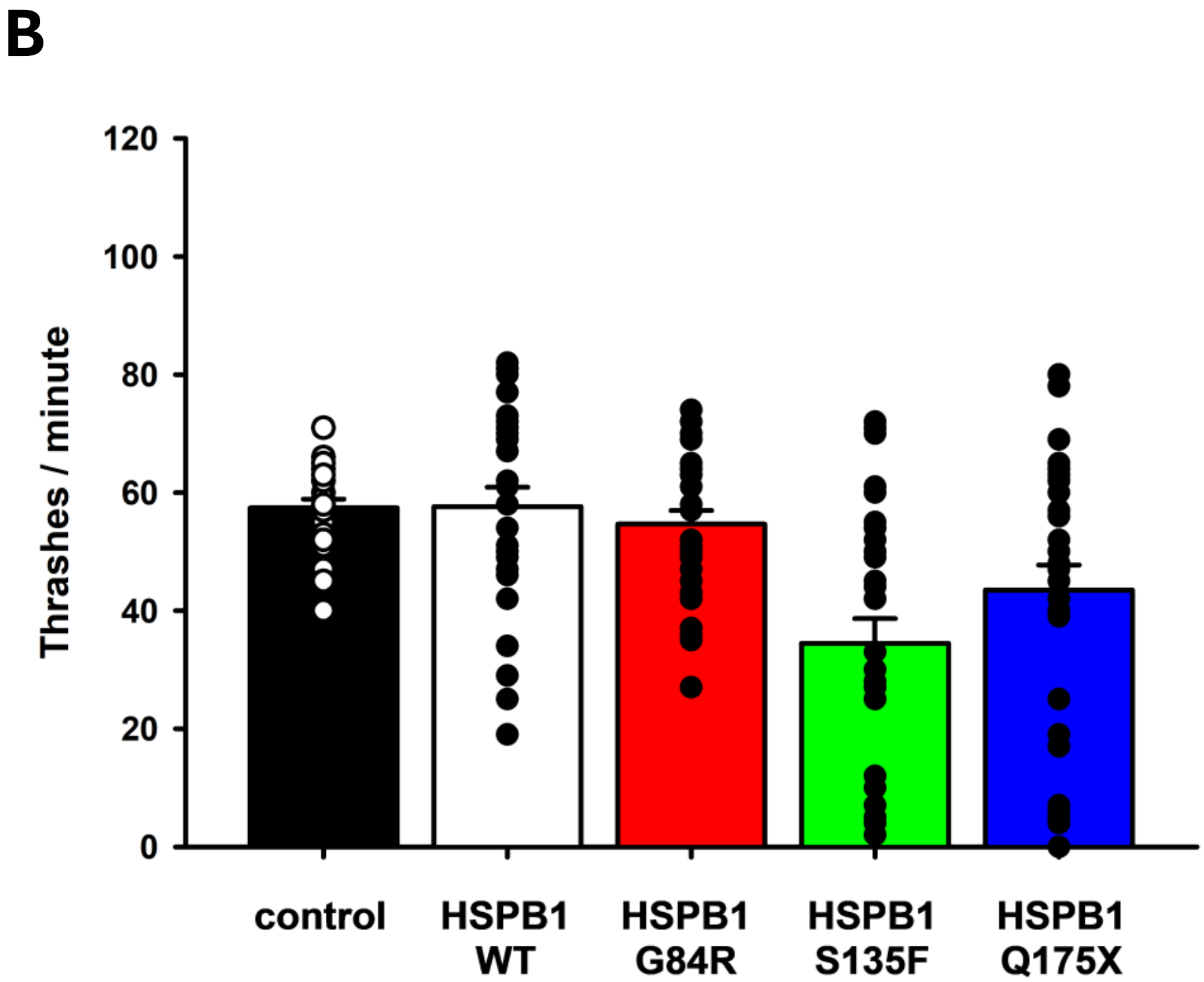

Disease Models & Mechanisms • Supplementary information

Fig. S2. Effects of HSPB1 CMT mutations on unnormalised *C. elegans* locomotion at (a) day 5 and (b) day 9. Data from Bristol N2 controls were compared to worms expressing either WT HSPB1 or 3 distinct CMT mutations of HSPB1 (G84R, S135F or Q175X).

A

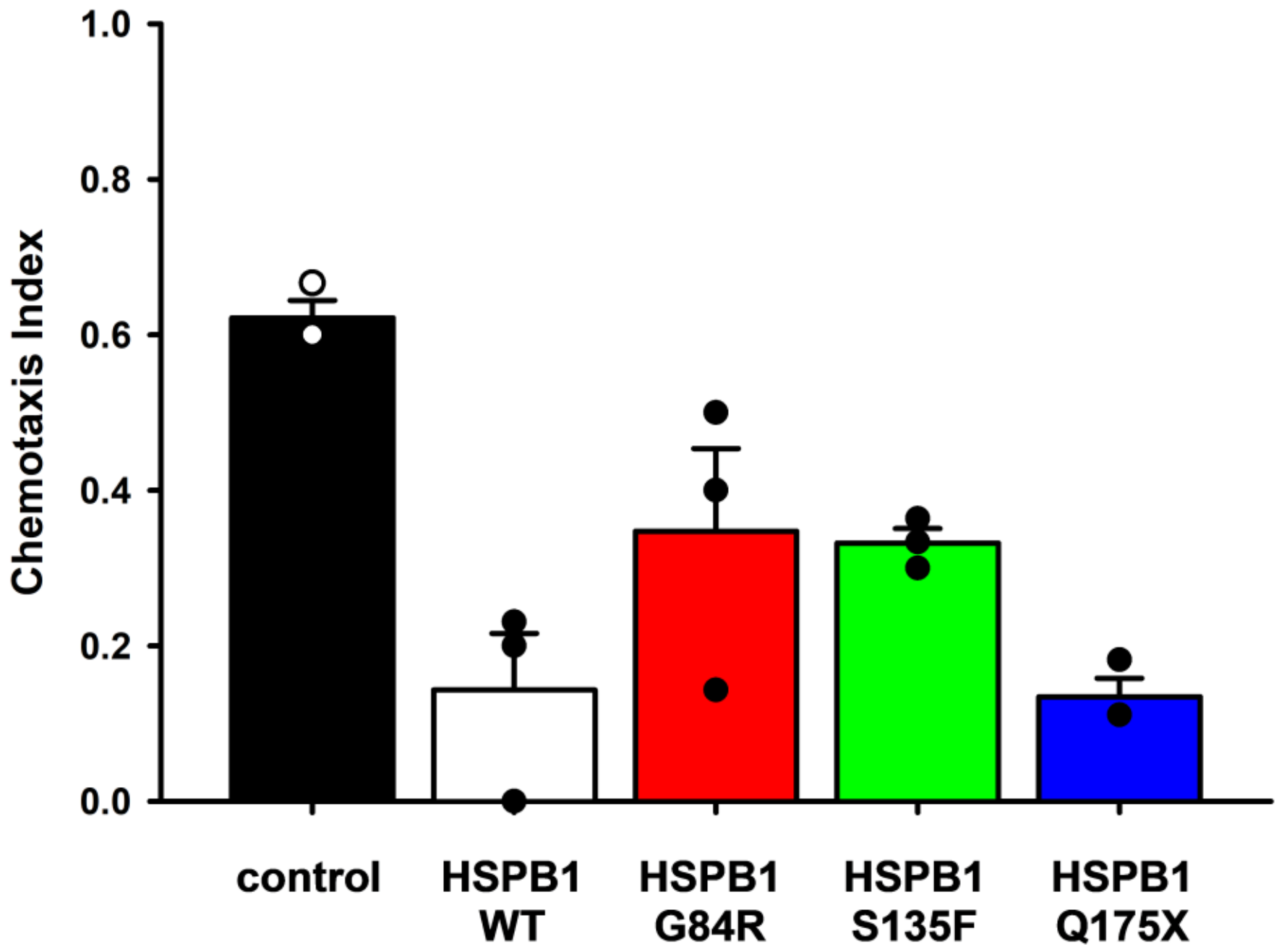

B

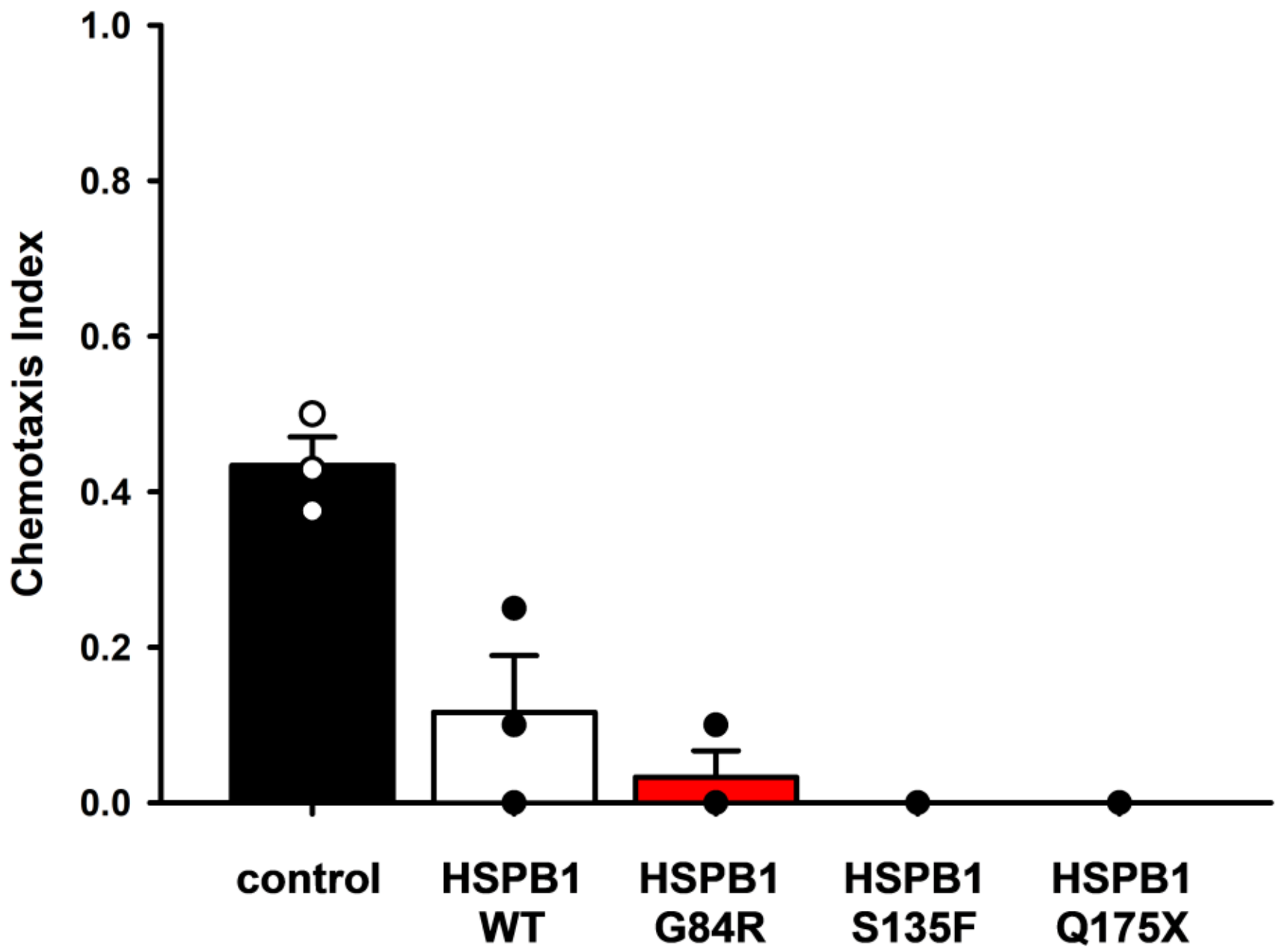

Disease Models & Mechanisms • Supplementary information

Fig. S3. Effects of HSPB1 CMT mutations on unnormalized *C. elegans* chemotaxis at (a) day 5 and (b) day 9. Data from Bristol N2 controls were compared to worms expressing either WT HSPB1 or 3 distinct CMT mutations of HSPB1 (G84R, S135F or Q175X).

Disease Models & Mechanisms • Supplementary information

A

```
HSPB1           MTE----RRVPFSLLRGPSWD-------PFRDW-----Y---PHS--RLF------DQAFGLPRLPEEWSQWLGGSSWPGYVRPLPPAA-----------------------IE------  64
hsp-12.1        ----------------------------------------------------------------------------------------M-----------------------HT------  3
hsp-12.2        ----------------------------------------------------------------------------------------M-----------------------SA------  3
hsp-12.3        ----------------------------------------------------------------------------------------------------------------MS------  2
hsp-12.6        ----------------------------------------------------------------------------------------M-----------------------MS------  3
hsp-16.1        ---------------------------------------------------------------------------------MSLYHYFR-----------------------PA---QRS  13
hsp-16.2        ---------------------------------------------------------------------------------MSLYHYFR-----------------------PA---QRS  13
hsp-16.20       ---------------------------------------------------------------------------------MSVNPFFD-----------------------PR---TLG  13
hsp-16.21       ---------------------------------------------------------------------------------MSVSPFFN-----------------------PK---RDS  13
hsp-16.41       -------------------------------------------------------------------------------MLMLRSPYSD-----------------------SN---ALD  15
hsp-16.48       -------------------------------------------------------------------------------MLMLRSPFSD-----------------------SN---VLD  15
hsp-17          --------------------------------------------------------------------------------MDRRFPPFS-----------------------PFFNHGRR  17
hsp-25          ----------MPTYTRTYSYSKREMSERRIDVNRSNYSVIDNEFGNMRDRFEQEMRRVEEEMKRLRSEFEGYRPNGGPPAAISNQPYNAYSNTSSHHETSNRTGGFGSPLPPPSFHGPSD  110
hsp-43          MTL----ATRHADFTRDKLWKENDLWLDDFRDW-----PLDWPKP--RDF------FHRFSR-DVDSWWKDWP--TDWPRMDAVMPRFS-----------------------SQLDRMDR  77
sip-1           --------------------------------------------------------------------------------MSSLCPYTG-----------------------R---PTG  13
Y55F3BR.6       ----------MATFNTTSSYN------RTYEKK-----VIEEGSPNVRVTTQTH--TAIPGMPDLGNIHS---------SMLSNFPNLA-----------------------IS------  59
ZK1128.7        MHHVDFDDDDLDSYLEGSVWSEKPSASEIFS-----------SKP--GDV------ISVFPRSSHSNMIYGYP--INY--KETVFPTHR-----------------------GP---PDQ  71

HSPB1           ------------SPAVAAPAYSRALSRQL--SSGVSEIRHTADRWRVSLDVNHFAPDELTVKTKDGVVEITGKHEERQDEHGYISRCFTRKYTLPPGVDPTQVSSSLSPEGTLTVEAPMP  170
hsp-12.1        ------------IPITTDSAASWDWPLQH--NDGVVKVTNTSEKFEVGLDAGFFGPNDIDVKVNGIEIIIHLRHDNRPTEYGIVNREVHRTYKLPEDVDPSTVRSHLNSSGVLTITANKL  109
hsp-12.2        ------------IEVTADAASTWDWPLQH--NDGVVKVHNTKEKFEVGLDVQFFTPKEIEVKVSGQELLIHCRHETRSDNHGTVAREINRAYKLPDDVDVSTVKSHLATRGVLTITASKK  109
hsp-12.3        ------------VAIDHDQTAKWDWPFQK--GDGVVKVLDYEDHFEVGLEAHNFLPNEIDVKNIGEFLEIHMAHTTKDDKFGSITRSITRCYRLPKGTDPATIKSKLDGSGILHISGNKK  108
hsp-12.6        ------------VPVMADEGTKWDWPLQK--GDGVVNVLDDDDHFEVGLEAHNFLPKEIEVKNIGELLEIHMEHNVKKDSFGDVSRNITRCYKLPKNVDMKTIKSNLDSHGILHIEARKM  109
hsp-16.1        VFGDL-MRDMAQMERQFTP--V-CRG----SPSESSEIVNNDQKFAINLNVSQFKPEDLKINLDGHTLSIQGEQ-ELKTEHGYSKKSFSRVILLPEDVDGAVASNLSEDGKLSIEAPKK  124
hsp-16.2        VFGDL-MRDMALMERQFAP--V-CRI----SPSESSEIVNNDQKFAINLNVSQFKPEDLKINLDGRTLSIQGEQ-ELKTDHGYSKKSFSRVILLPEDVDGAVASNLSEDGKLSIEAPKK  124
hsp-16.20       EMV---FGDNGRLDREYVP--I-SENN-DDLSDCRNEIVDTHEKFSVNLNVPDVKPEELKINLEGRKLSIKAEHQEIENDNISTTQTYSKSIVLPEDVDVTHLSSNLSEDGKLLIEVPKV  126
hsp-16.21       QLGEM-MRDMGGMQRRLMP--I-SGTF--NPMTDDSEIMNSNDKFAVNLNVSNFKPEELKVNLEGRQLSIQGEH-DVENEHGASRKSFSRMILLPEDVDITSVATNLSNDGKLCIEAPKL  126
hsp-16.41       HFLDE-LTGSVQFPYWRNA--D-HNSF--NFSDNIGEIVNDESKFSVQLDVSHFKPENLKIKLDGRELKIEGIQ-ETKSEHGYLKRSFSKMILLPEDADLPSVKSAISNEGKLQIEAPKK  128
hsp-16.48       HFLDE-ITGSVQFPYWRNA--D-HNSF--NFSDNIGEIVNDESKFSVQLDVSHFKPEDLKIELDGRELKIEGIQ-EKKSEHGYSKRSFSKMILLPEDVDLTSVKSAISNEGKLQIEAPKK  128
hsp-17          FFDDVDFDRHMIRPYWADQTML-----TGHRVGDAIDVVNNDQEYNVSVDVSQFEPEELKVNIVDNQLIIEGKHNEKTDKYGQVERHFVRKYNLPTGVRPEQIKSELSNNGVLTVKYEKN  132
hsp-25          LMA--------HRP-T-------YDPYLDNLKSPLIKDESDGKTLRLRFDVANYKPEEVTVKTIDNRLLVHAKHEE-KTPQRTVFREYNQEFLLPRGTNPEQISSTLSTDGVLTVEAPLP  213
hsp-43          NWR--------SDPYWMNLYPRWAEPIFKEGIDVNSNVVNDDRRFAVDMDCYQFRPEEIQVKTLDDTLMIEGRHEDIRDKDNFTKMYFVRKYQLPRDVDFNSIQSSIDAKGRLQVEAGKF  189
sip-1           LFRD--FED--MMPYWAQRHSM-LNNFNNIVPQQLNEVENTAQKFCVKLDVAAFKPEELKVNLEGHVLTIEGHH-EVKTEHGFSKRSFTRQFTLPKDVDLAHIHTVINKEGQMTIDAPKT  127
Y55F3BR.6       ------------S-----------PSVVGSQNGNLTSIRVTNTSFHAILDVSKYDADSLKVTVVDNNIIVEGSHGEKEDTYGTIESTFKRRFPLPKAVAPESVQSQLTADGHLTIDAKAP  156
ZK1128.7        SY----------DSYYTKIT--ESRPRSPGPVAGAGEITNTSHGFTIEIDVFHFMPEEIKVVLTDDTLSISGERFESTGDGHTLRRSFSRKYSIPDDVHLDTIRSHLTNSGVLIINGSRK  179
                                                    .           .:      ..: :   .  : :   :               :   :*  .    : : :   * : :

HSPB1           KLA-TQSNEITIPVT----FESRA----Q------LGGPEAAKSDETAAK----------------------------------------------------------------------  205
hsp-12.1        ------------------------------------------------------------------------------------------------------------------------  109
hsp-12.2        A-----------------------------------------------------------------------------------------------------------------------  110
hsp-12.3        K-----------------------------------------------------------------------------------------------------------------------  109
hsp-12.6        H-----------------------------------------------------------------------------------------------------------------------  110
hsp-16.1        EAIQ----GRSIPI----QQAPVEQKTSE-------------------------------------------------------------------------------------------  145
hsp-16.2        EAVQ----GRSIPI----QQAIVEEKSAE-------------------------------------------------------------------------------------------  145
hsp-16.20       EAKK----TNFFGFLSKFRCMPESQ-----------------------------------------------------------------------------------------------  147
hsp-16.21       EGVC----GRSVPV----KEASMDHHHIE-------------------------------------------------------------------------------------------  147
hsp-16.41       TN-S----SRSIPI----NFVAKH------------------------------------------------------------------------------------------------  143
hsp-16.48       TN-S----SRSIPI----NFVAKH------------------------------------------------------------------------------------------------  143
hsp-17          QEQQP----KSIPIT----IVPKRN-----------------------------------------------------------------------------------------------  149
hsp-25          QLAIQQ------------------------------------------------------------------------------------------------------------------  219
hsp-43          NNMALQGRERMIPIE----GAGHHSPRFENGTLRSQRGPNSPIHVQTEHDGRSVSSRSGSRLNDSPGSRDVYSSHSYSYHRSDSRNRLSPNDVNITRNDNRTYSPVTPRITTSGSYNTAG  305
sip-1           GSNTT---VRALPIHTSAGHAVTQKPSSTTTTGKH-------------------------------------------------------------------------------------  159
Y55F3BR.6       EPKQEGARPIQIKVI----NTSAEQQK-Q-------------------------------------------------------------------------------------------  180
ZK1128.7        GWRETSISS-YHPTT-------------QRNVARSVTS----IV----------------------------------------------------------------------------  205

HSPB1           ---------------------------------------------------------------  205
hsp-12.1        ---------------------------------------------------------------  109
hsp-12.2        ---------------------------------------------------------------  110
hsp-12.3        ---------------------------------------------------------------  109
hsp-12.6        ---------------------------------------------------------------  110
hsp-16.1        ---------------------------------------------------------------  145
hsp-16.2        ---------------------------------------------------------------  145
hsp-16.20       ---------------------------------------------------------------  147
hsp-16.21       ---------------------------------------------------------------  147
hsp-16.41       ---------------------------------------------------------------  143
hsp-16.48       ---------------------------------------------------------------  143
hsp-17          ---------------------------------------------------------------  149
hsp-25          ---------------------------------------------------------------  219
hsp-43          NANLHEERSSSRAQSHRSESRNGGYRVESPVSTTTGILRNGNSDSPNSTQREYRSIQILRKTY  368
sip-1           ---------------------------------------------------------------  159
Y55F3BR.6       ---------------------------------------------------------------  180
ZK1128.7        ---------------------------------------------------------------  205
```

B

```
HSPB1           MTERRVPFSLLRGPSWDPFRDWYPHSRLFDQAFGLPRLPEEWSQWLGGSSWPGYVRPLPPAAIESPAVAA---PAYSRALSRQLSSGVSEIRHTADRWRVSLDVNHFAPDELTVKTKDGV  117
hsp-16.1        ------------------------------------------------MSLYHYFRPAQRSVFGDLMRDMAQMERQFTPVCRGSPSESSEIVNNDQKFAINLNVSQFKPEDLKINLDGHT  72
hsp-16.48       -------MLMLRS------------------PFSDSNVLDHFLDEITGSVQFPYWRNADHNS-------------------FNFSDNIGEIVNDESKFSVQLDVSHFKPEDLKIELDGRE  76
                                                                     * *                             .  .** :  .:: :.*:*.:* *::*.:: ..

HSPB1           VEITGKHEERQDEHGYISRCFTRKYTLPPGVDPTQVSSSLSPEGTLTVEAPMPKLATQSNEITIPVTFESRAQLGGPEAAKSDETAAK  205
hsp-16.1        LSIQGEQ-ELKTEHGYSKKSFSRVILLPEDVDGAVASNLSEDGKLSIEAPKKEAIQ---GRSIPIQQAPVEQKTSE-----------  145
hsp-16.48       LKIEGIQ-EKKSEHGYSKRSFSKMILLPEDVDLTSVKSAISNEGKLQIEAPKKTNS----SRSIPINFVAKH----------------  143
                :.* * : * : **** .:.*::   ** .**   * * :* :*.* :***           :**:
```

Disease Models & Mechanisms • Supplementary information

Fig. S4. Sequence alignment of human HSPB1 with *C. elegans* small heat shock proteins (sHSPs). (A) Alignment of HSPB1 with all alpha-crystallin containing proteins in the *C. elegans* genome. As is seen with most sHSPS, the alpha-crystallin domains are relatively conserved, whereas the N- and C-termini are variable. (B) Alignment of HSPB1 with the proteins examined in this paper. The HSP-16.11 and HSP-16.49 proteins (not shown) have identical primary amino acid sequences to HSP-16.1 and HSP-16.48. All alignments were made by multiple sequence alignments using Clustal Omega (Madeira et al., 2024). * equals identical amino acid, . or : indicates similar amino acids.

Disease Models & Mechanisms • Supplementary information

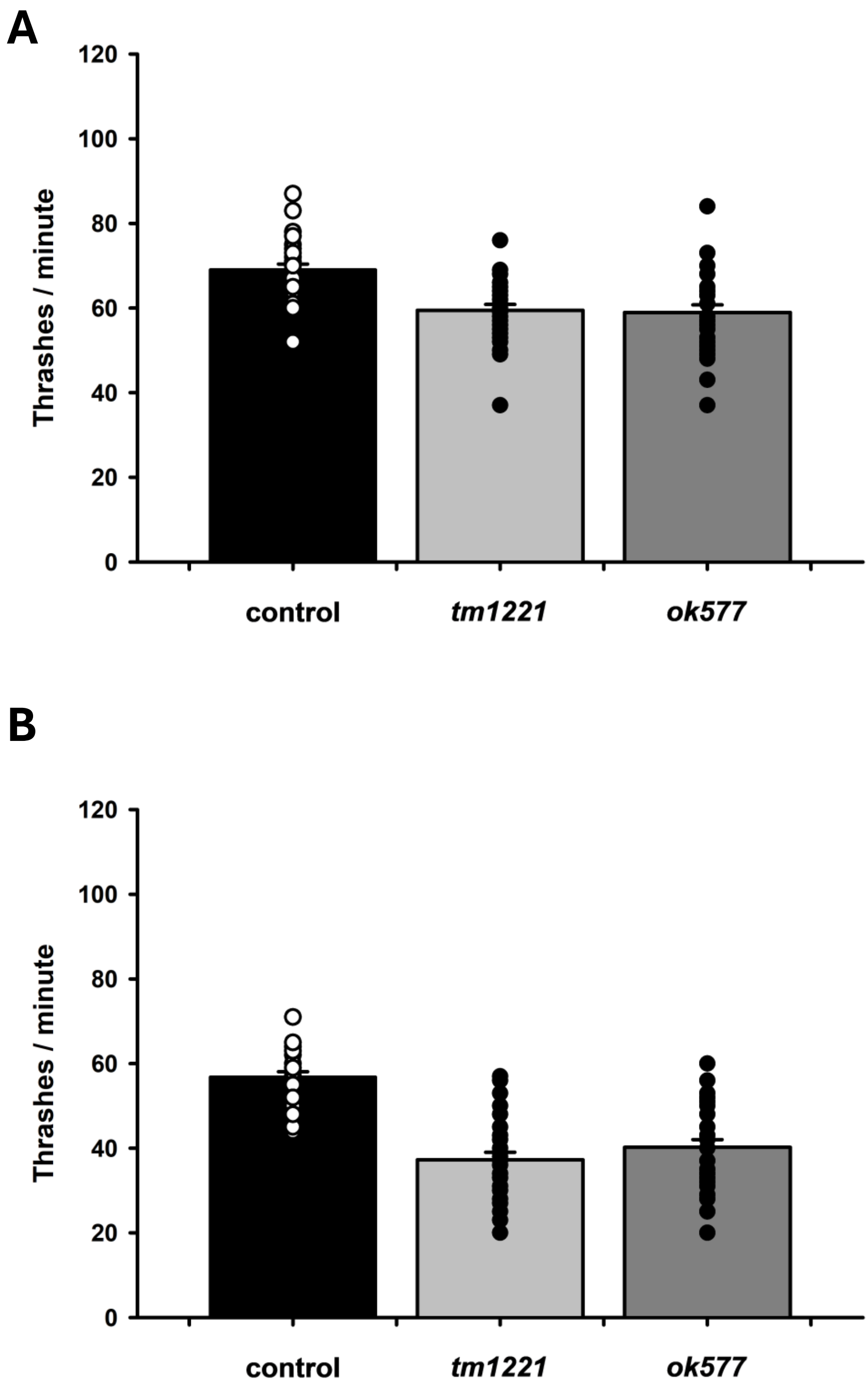

Fig. S5. Effects of sHSP mutation on unnormalised *C. elegans* locomotion at (a) day 5 and (b) day 9. Data from Bristol N2 controls were compared to either the *ok577* allele (deletion mutation for genes *hsp-16.1/hsp-16.48*) or the *tm1221 allele* (deletion mutation for genes *hsp-16.11/hsp-16.49*).

Disease Models & Mechanisms • Supplementary information

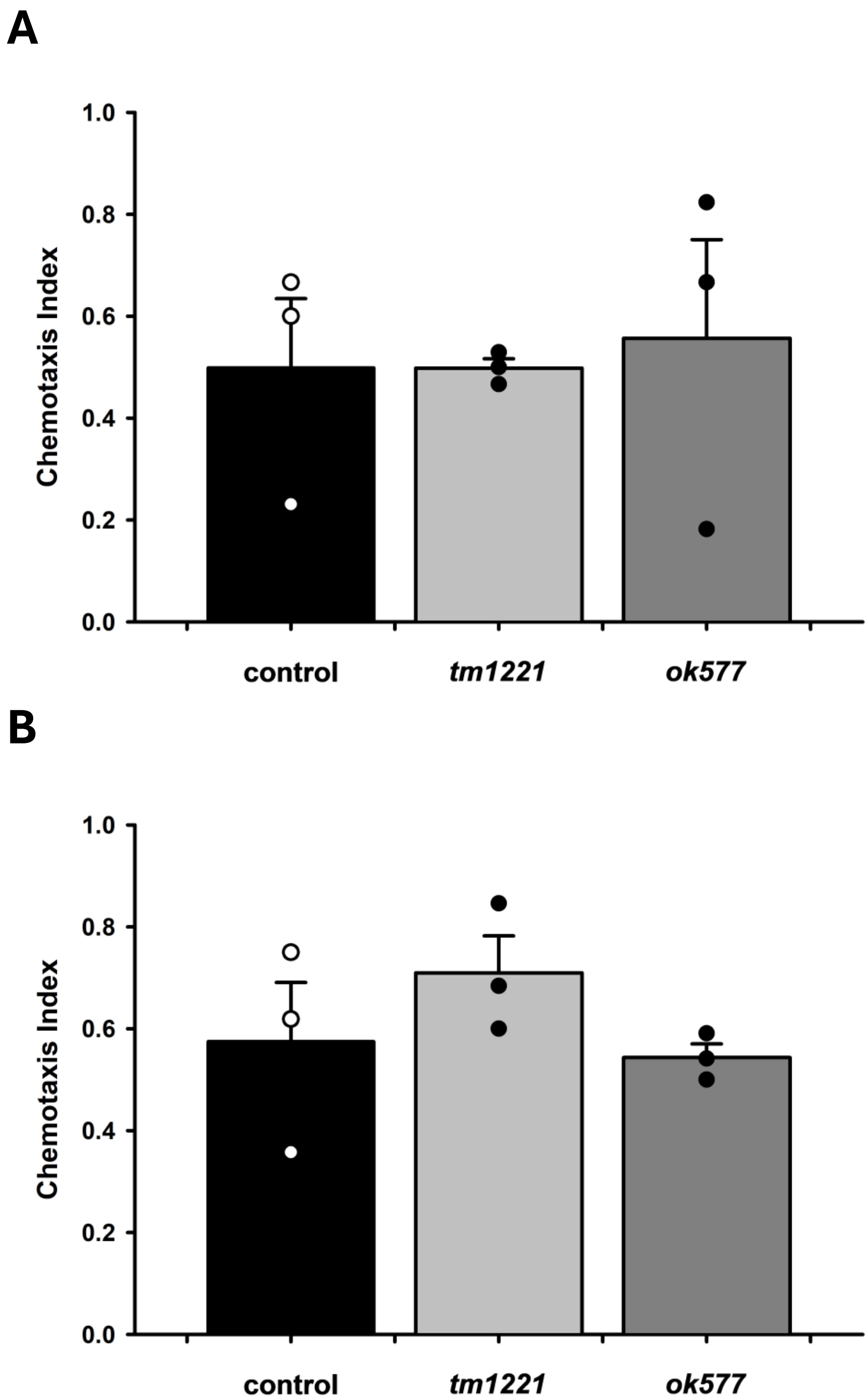

Fig. S6. Effects of sHSP mutation on unnormalized *C. elegans* chemotaxis at (a) day 5 and (b) day 9. Data from Bristol N2 controls were compared to either the *ok577* allele (deletion mutation for genes *hsp-16.1/hsp-16.48*) or the *tm1221 allele* (deletion mutation for genes *hsp-16.11/hsp-16.49*).

Disease Models & Mechanisms • Supplementary information

Supplementary reference

Madeira, F., Madhusoodanan, N., Lee, J., Eusebi, A., Niewielska, A., Tivey, A. R. N., Lopez, R. and Butcher, S. (2024). The EMBL-EBI Job Dispatcher sequence analysis tools framework in 2024. *Nucleic Acids Res.* **52**, W521-W525. doi:10.1093/nar/gkae241
